# Modulation of macrophage survival during *Klebsiella pneumoniae* infections

**DOI:** 10.3389/fcimb.2026.1757662

**Published:** 2026-02-25

**Authors:** Nithya N., Anjoom Mohamed Ali, Sruthi K. P., Subhash Mehto, Cecil Antony

**Affiliations:** 1 Infection Immunology Lab, National Institute of Technology Calicut, Department of Bioscience and Bioengineering, Kozhikode, India; 2 Immuno Biology Lab, Indian Institute of Technology, Dharwad, India

**Keywords:** innate immunity, macrophage, *Klebsiella pneumoniae*, infection, calcium homeostasis, survival

## Abstract

*Klebsiella pneumoniae*, a facultative anaerobe and Gram-negative bacterium, has high clinical significance in public health concerns. *K. pneumoniae* is given its name in recognition of the German-Swiss microbiologist Edwin Klebs. Carl Friedlander first identified *K. pneumoniae* as a bacterium isolated from the lungs of individuals who had died from pneumonia. *K. pneumoniae* is a prominent member of the *Enterobacteriaceae* family, an opportunistic pathogen, and one of the pathogens sweeping the world in the antibiotic resistance pandemic. This organism accounts for approximately one-third of all Gram-negative infections, including pneumonia, urinary tract infections, liver abscesses, and bacteremia. Classically, these infections commonly occur in individuals who are hospitalized or immunocompromised and regularly treated with *β*-lactams along with other antibiotics effective against *Enterobacteriaceae*. Unfortunately, the emergence of multidrug-resistant infections in patients treated at intensive care units (ICUs) has been a serious problem faced by clinicians globally. *K. pneumoniae* has been recently added to the critical list of microorganisms by the World Health Organization (WHO), highlighting the urgent requirement for new therapeutic options to combat this bacterium. Developing new therapeutic interventions requires a comprehensive understanding of host-pathogen interactions and the role of immune cells in clearing the bacteria. To that end, macrophages play a crucial role in safeguarding our body against infectious agents, as well as contributing to processes such as pathogen clearance and tissue homeostasis. Calcium, a secondary messenger, refines the macrophage protective responses and influences cell survival. Many pathogens have evolved tactics to circumvent the innate immune clearance mechanisms exerted by macrophages. Therefore, this review will focus on the modulation of macrophage innate immune responses and survival upon *K. pneumoniae* infection.

## 1 Introduction

Macrophages are a crucial component of the innate immune system, serving as the first line of defense against microbial pathogens and key regulators of immune homeostasis (Chen et al., 2023). These versatile immune cells exhibit remarkable plasticity and functionality, capable of recognizing, engulfing, and eliminating pathogens while modulating the immune response through the secretion of cytokines and chemokines (Brancewicz et al., 2025). The survival and functional integrity of macrophages are central to mounting an effective immune response, particularly during bacterial infections. However, the interaction between macrophages and bacterial pathogens is often characterized by a complex interplay of host defense mechanisms and microbial strategies aimed at immune evasion (Pandey et al., 2022). Among bacterial pathogens, *K. pneumoniae* has emerged as a formidable global health threat due to its multidrug resistance and high virulence. Its association with severe nosocomial as well as community-acquired infections, such as pneumonia, bloodstream infections, and urinary tract infections, confirms its vast incidence (Mohd Asri et al., 2021).

Despite extensive research into its virulence factors, the mechanisms by which *K. pneumoniae* modulates host immune responses, particularly macrophage survival and function, remain completely grey. Interestingly, the pathogen employs various strategies, including the prevention of phagocytosis, avoidance of autophagic pathways, and modulation of host cell death processes, to evade clearance and to establish infection. Undoubtedly, evasion of macrophage innate immune defense mechanisms not only promotes bacterial survival but also leads to immune dysregulation, resulting in chronic systemic infections. Especially, *K. pneumoniae* has been recognized for evading host immunity, and also displayed increased resistance to multiple antimicrobial drugs, including third-generation antimicrobials (i.e., Tigecycline, colistin, and carbapenem) (Abhinand et al., 2025). Despite several studies being conducted to understand the biology of *K. pneumoniae* infections worldwide, a significant gap remains. Unfortunately, the interaction of *K. pneumoniae* with the human immune system has not yet been fully understood.

More importantly, calcium signaling, a regulator of cellular homeostasis and immune responses, has garnered increasing attention for its role in regulating macrophage survival and immunity. Calcium plays a pivotal role in processes such as phagocytosis, cytokine production, and the regulation of cell death (Nunes and Demaurex, 2010). This is an integrative review of the mechanisms, including the most recent discoveries, by which *K. pneumoniae* infections influence macrophage survival, function, and immune regulation.

## 2 Overview of macrophage homeostasis

Macrophages, first described by Metchnikoff during his investigations into primitive organisms devoid of adaptive immunity, are among the oldest cell types in metazoan evolution. Primarily recognized for their phagocytic capabilities, Metchnikoff discovered that macrophages play a crucial role in maintaining homeostasis through tissue resorption and nutrient acquisition, in addition to serving as primary defenders against invading pathogens (Metchnikoff, 1893). His pioneering research into phagocytosis laid the foundation for our present-day understanding of cellular immunity and the structural basis of host defense (Mechnikov, 1988). Macrophages found in vertebrate tissues form a network of vast and dynamic organ systems that arise during mid-gestation and continue throughout life (Gordon and Plüddemann, 2017). They exhibit structural and functional variation in adult animals by building a complex three-dimensional network in tissues (Wynn et al., 2013). As immune sentinels, macrophages are the first line of defense against bacteria, conferring protection to the host (Joshi and Saroj, 2023).

### 2.1 Origins and development of macrophages

**Figure 1:**
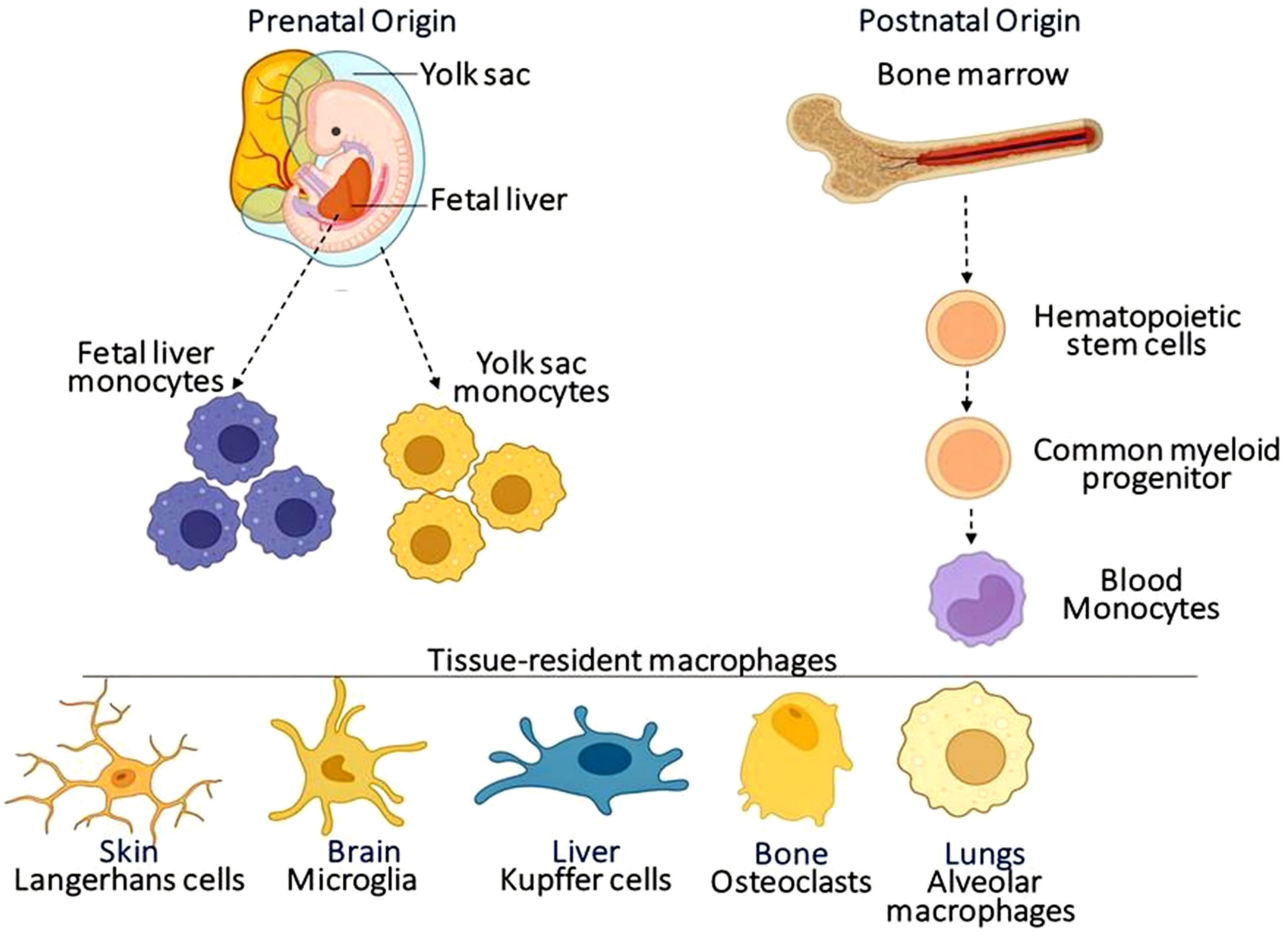
Origin and classification of macrophages. Tissue-resident macrophages arise from bone marrow-derived monocytes as well as from the yolk sac and fetal liver. In some instances, tissue-resident macrophages might be replenished by blood monocytes. Different tissue-resident macrophages were known by different names (Langerhans cells, Microglia, Kupffer cells, Osteoclasts, and Alveolar macrophages).

Macrophages originate from distinct developmental pathways during embryogenesis and continue to adapt and renew throughout life (Gautier et al., 2012) as summarized in **Figure 1**. Macrophage origin, migration, and development have high similarity between humans and mice (Guan et al., 2025). In early embryonic development, macrophages arise from the yolk sac independently of hematopoietic stem cells (HSCs). These yolk sac-derived macrophages, such as microglia in the brain, establish in tissues and persist throughout adulthood without significant input from circulating monocytes. This discovery has overturned the previous dogma of macrophage ontogeny, revealing that tissue-resident macrophages are not exclusively derived from bone marrow progenitors as previously believed (Mosser et al., 2021; Ginhoux and Jung, 2014). A second wave of progenitors is erythro-myeloid precursors (EMPs) originating from the yolk sac. These EMPs subsequently migrate to the developing fetal liver, where they give rise to macrophages that seed tissues such as the liver and spleen (Bian et al., 2020).

In contrast to yolk sac-derived macrophages (like microglia), which are largely self-renewing, these populations have a greater dependence on circulating monocytes for maintenance (Hirayama et al., 2017). Postnatally, HSCs in the bone marrow continuously produce monocytes, which can develop into macrophages in response to local signals, especially in high-turnover tissues like the gut or during inflammatory conditions (Gordon and Plüddemann, 2017). The tri-lineage origin, comprising the yolk sac, EMPs, and HSCs, demonstrates the complexity of macrophage development and emphasizes their specialized roles in tissue homeostasis. Tissue-resident macrophages maintain their populations primarily through self-renewal under steady-state conditions, whereas monocyte-derived macrophages are recruited and contribute to inflammation or injury (Guan et al., 2025).

### 2.2 Functional heterogeneity and plasticity of macrophages

**Figure 2:**
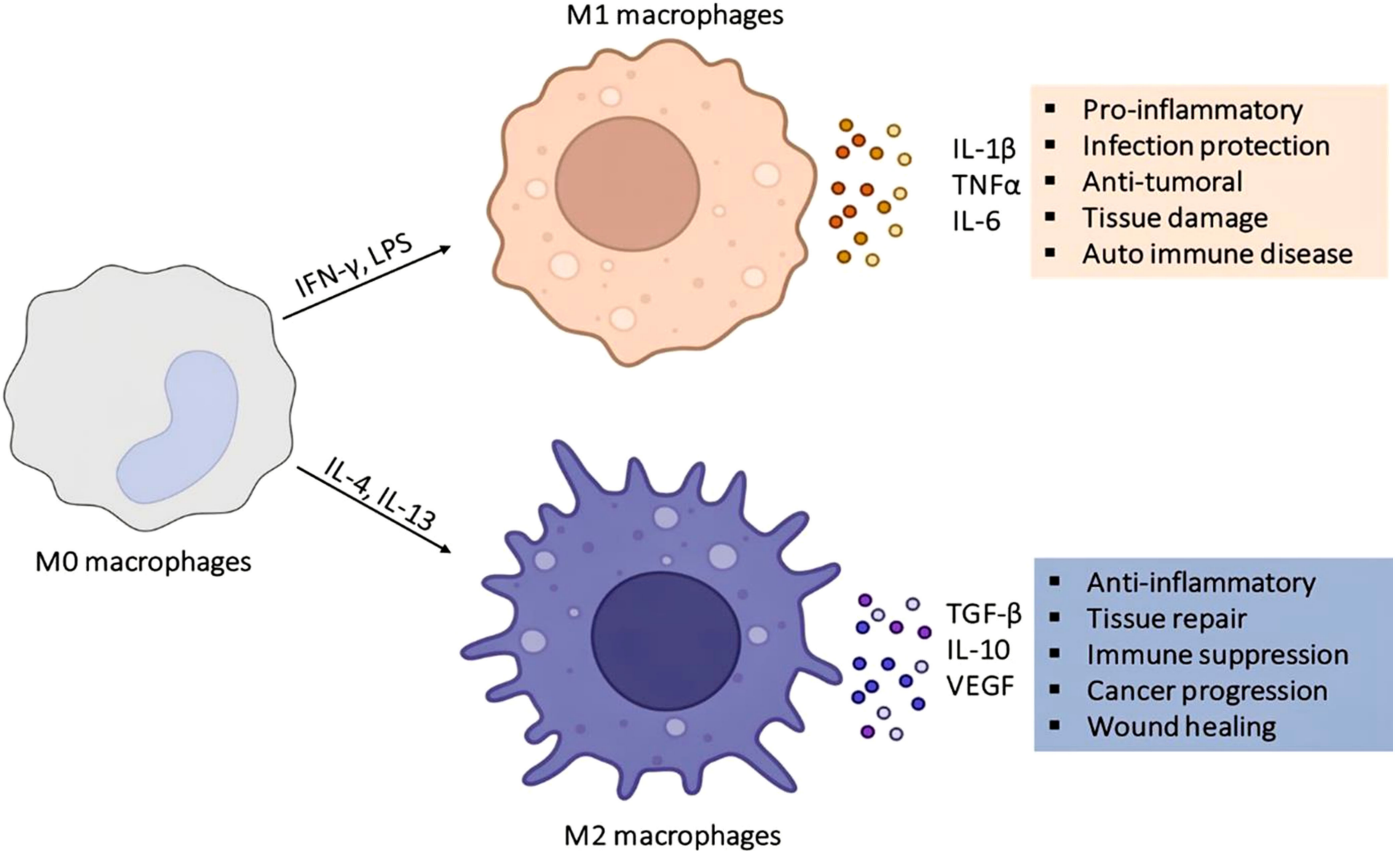
M1 and M2 polarization of macrophages. Pro-inflammatory M1 polarization and anti-inflammatory M2 polarization of macrophages. IFN-γ: Interferon gamma, LPS: lipopolysaccharide treatment of Mo macrophages leads to M1 polarization. The induction of interleukin-4 (IL-4) and Interleukin-13 (IL-13) in macrophages leads to M2 polarization. M1 macrophages secrete IL-1beta, Tumor Necrosis Factor (TNF), IL-1beta, IL-6) like pro-inflammatory cytokines. Conversely, M2 macrophages secrete Transforming Growth Factor (TGF), vascular endothelial growth factor (VEGF), and Interleukin-10 (IL-10), which are anti-inflammatory cytokines.

Macrophages exhibit incredible plasticity and functional diversity, enabling them to adapt to the needs of their microenvironments. This plasticity enables them to perform diverse functions, such as pathogen clearance, tissue repair, immune surveillance, and metabolic regulation (Chi et al., 2025). Macrophages exhibit a range of tissue-specific roles, resulting in a variety of different phenotypes. For instance, the Kupffer cells in the liver regulate lipid metabolism and detoxify harmful substances (Murray and Wynn, 2011). Similarly, alveolar macrophages in the lungs play an important role in maintaining surfactant homeostasis and providing defense against microbes (Hirayama et al., 2017). Macrophages in the bone, known as osteoclasts, are responsible for bone matrix resorption, and microglia in the brain are involved in neural development and maintenance. In general, these tissue-specific functions are driven by local cues, such as cytokines, metabolites, and growth factors, which initiate different transcriptional pathways (Gordon and Plüddemann, 2017). Macrophages demonstrate remarkable adaptability induced by inflammatory stimuli, which dictates their phenotype. For instance, classically activated macrophages (M1) are characterized by the production of pro-inflammatory cytokines and molecules that help combat invading microbial pathogens. On the contrary, alternatively activated macrophages (M2) produce anti-inflammatory cytokines and growth factors that promote the tissue repair process (Luo et al., 2024), as shown in **Figure 2**, which depicts the polarization of macrophages. However, this traditional paradigm oversimplifies the wide and dynamic phenotypic spectrum of macrophage activation states observed *in-vivo*, which are influenced by the specific microenvironment (Mosser et al., 2021).

### 2.3 Mechanism of tissue homeostasis

Reducing macrophages to their immune functions alone ignores and overshadows their ancillary biological role. Furthermore, macrophages play a major role in host defense, together maintaining tissue integrity and physiological homeostasis (Mosser et al., 2021). The cellular debris, immune complexes, apoptotic cells, pathogens, and other waste materials are scavenged by macrophages to prevent any disruption in organ function. This proficient phagocytic activity highly facilitates the recycling of cellular components while supporting tissue health (Hirayama et al., 2017). Macrophages also play a significant role in key metabolic pathways, including the turnover of iron, Ca^2+^, lipids, and amino acids, and bilirubin, upholding the maintenance of homeostatic levels of these molecules (Kosteli et al., 2010; Biswas and Mantovani, 2012; Ganz, 2016; Roodman, 1999; Suda et al., 1992). However, the growth factors or signaling molecules secreted by macrophages govern tissue remodeling, wound repair, organogenesis, angiogenesis, and metabolic regulation. Therefore, the multi-angled activities highlight macrophages’ dual role in host defense and tissue homeostasis (Brancewicz et al., 2025; Ferrante and Leibovich, 2012; Guan et al., 2025).

Exclusively, the Colony-Stimulating Factors (CSFs) play a pivotal role in macrophage homeostasis, balancing survival, proliferation, and differentiation processes. Macrophage CSF1 and its receptor (CSF1R) are essential for the survival and self-renewal of macrophages. More importantly, brain tissue-resident macrophages, i.e., microglia, possess an alternative ligand for CSF1R (i.e., IL-34), which has been reported to be important to the localized maintenance of tissue-resident macrophages (Xiang et al., 2023). The identity and plasticity of macrophages are additionally confirmed by transcriptional regulators like PU.1, the master transcriptional factor controlling the expression of macrophage-specific genes (Wynn et al., 2013). However, transcription factors such as MafB and GATA6 are reported to be important for the tissue-specific differentiation of macrophages (Hamada et al., 2020). Consequently, the transcriptional networks involving these factors allow macrophages to rapidly respond and adapt to physiological changes. In fact, in the brain and liver tissues with minimal macrophage turnover rate, there exists a self-renewal mechanism to replenish the resident macrophage population (Röszer, 2018). Conversely, in the gut tissues with a high macrophage turnover rate, continuous replenishment of macrophages happens from monocyte-derived precursors to restore their numbers (Viola and Boeckxstaens, 2021). Therefore, this remarkable adaptability of tissue-specific macrophage homeostasis has been lacking in other similar immune cell types.

### 2.4 Survival mechanisms

Macrophage survival is closely associated with their ability to detect and respond to environmental signals. Cytokines like IL-10 and Transforming Growth Factor beta (TGF-β) provide anti-apoptotic signals that promote macrophage survival, particularly during tissue repair and the resolution phase of inflammation. Additionally, these cytokines also skew the macrophages toward anti-inflammatory phenotypes, ensuring that they support tissue homeostasis (Yan et al., 2024). Metabolic reprogramming is important for macrophage survival, where the classically activated (M1) macrophages rely predominantly on glycolysis to maintain their pro-inflammatory nature, while the alternatively activated (M2) macrophages use oxidative phosphorylation and fatty acid oxidation to fulfil their reparative activities. These distinct metabolic changes allow macrophages to meet the energy and biosynthetic needs of their functional roles (Wang et al., 2022). Efferocytosis, the process by which macrophages phagocytize apoptotic cells, also provides survival signals. By removing cellular debris, macrophages not only prevent tissue damage but also obtain nutrients and growth factors that bolster their homeostatic functions. This process is important for maintaining tissue integrity and alleviating inflammation (Cabrera and Makino, 2022).

#### 2.4.1 Regulators of survival and cellular resilience

Macrophage Colony-Stimulating Factor (M-CSF) is required for macrophage survival. It binds to the M-CSF Receptor (CSF1R) and thereby activates downstream signaling pathways, including Phosphoinositide 3-Kinase (PI3K)-AKT and MAPK. These pathways help in cellular proliferation and prevent apoptosis by upregulating survival genes. Similarly, other cytokines such as Tumor Necrosis Factor Alpha (TNF-α) and Interleukin (IL)-6 act in an autocrine or paracrine manner to enhance macrophage survival during immune responses (Han et al., 2022). At sites of inflammation, cytokine gradients influence the survival and functionality of macrophages. For example, Interferon-γ (IFN-γ) increases macrophage responsiveness to pathogen-associated molecular patterns (PAMP), whereas IL-10 and IL-4 support the survival of tissue-resident macrophages in homeostatic conditions (Arora et al., 2018). Macrophage survival is greatly affected by the extracellular matrix (ECM) and the interactions within tissue environments. ECM components, like decorin, provide structural support and deliver biochemical signals that enhance macrophage viability. Notably, decorin binds to macrophage receptors, which helps to block apoptotic processes (Xaus et al., 2001).

Macrophages also communicate with other immune cells, such as lymphocytes and neutrophils. Consequently, these interactions ensure macrophage persistence in immune niches and their ability to mediate adaptive immune responses (Huang et al., 2025). Macrophages utilize cell cycle checkpoints to maintain a balance between survival and activation. IFN-γ, a cytokine that plays a role in macrophage activation, promotes the expression of cyclin-dependent kinase (CDK) inhibitors such as p21waf1. However, this induction causes halts in cell cycle progression at the G1/S phase, allowing macrophages to focus on their immune activities rather than proliferation. Therefore, the checkpoint strategy not only prepares macrophages for activation but also protects them from apoptosis triggered by inflammatory signals like lipopolysaccharides (LPS) (Xaus et al., 2001). The NF-κB (Nuclear Factor kappa-light-chain-enhancer of activated B cells) signaling pathway has been an important regulator for macrophage survival. Multiple mechanisms involving inflammation, immunity, and cell survival are controlled by this key transcription factor (Mussbacher et al., 2023).

In general, NF-κB resides in the cytoplasm as inactive forms by the inhibitory proteins (IκBs), preventing its translocation to the nucleus. External stimuli like cytokines, microbial products, or stress activate the IκB kinase (IKK) complex, leading to phosphorylation of IκBs, labelling them for ubiquitination and subsequent proteasomal degradation. Moreover, the translocation of NF-κB, into the nucleus targets transcription of genes, including anti-apoptotic proteins, cytokines etc. Indeed, those environments that promote inflammation, oxidative stress, and apoptosis genes are regulated by NF-κB, supporting macrophage resilience. Unfortunately, any accidental disruption of this pathway might lead to chronic inflammation or immune suppression (Dorrington and Fraser, 2019). The PI3K-AKT pathway is also an important regulator for macrophage survival, especially during stressed conditions such as infection (De-Leon-Lopez et al., 2023). When stimulated by growth factors such as cytokines, or PAMPs, PI3K phosphorylates phosphatidylinositol-4,5-bisphosphate (PIP2) resulting in the production of phosphatidylinositol-3,4,5-trisphosphate (PIP3), which acts as a lipid second messenger.

Subsequently, PIP3 then recruits Akt to the plasma membrane, where it will undergo phosphorylation and be activated. Akt, once phosphorylated, can then phosphorylate downstream targets such as Bad and GSK3β, which inhibits apoptosis, promoting anti-apoptotic signaling and increased production of anti-apoptotic proteins like Mcl-1. Especially, multiple mechanisms of macrophages, such as phagocytic function, cytokine release, and survival, are enhanced by the PI3K-AKT pathway, while suppressing the NF-κB activation, emphasizing its role in macrophage viability (Linton et al., 2019; Wang et al., 1999). The activation of the PI3K/AKT and NFκB pathways depends on constitutive calcium influx, through a mechanism that involves the calmodulin/calmodulin kinase II (CaM/CaMKII) axis, coupling constitutive calcium influx to the activation of survival signaling (Tano and Vazquez, 2011). Calcium have role in macrophage cell signaling and biology, which determines the fate of innate immune responses. Therefore, we realized it is important to delineate calcium homeostasis in macrophages, which alters their survival and immune function.

## 3 Role of calcium in macrophage survival and immune regulation

Calcium (Ca^2+^), a secondary messenger, dictates cell survival and the immune responses against invading pathogens. A fundamental aspect of calcium signaling is that alterations in intracellular calcium concentration [Ca^2+^]_i_ elicit specific cellular responses (Berridge et al., 2000). Calcium entry and exit are governed by transmembrane channels that specifically allow the influx/efflux of Ca^2+^ ions based on the cellular demands. In unstimulated cells, the cytosolic calcium concentration [Ca^2+^]_i_ is maintained at ~100 nM, commonly referred to as the resting or basal calcium concentration (Bootman and Bultynck, 2020). Upon stimulation, cytosolic calcium levels rapidly increase from this resting state and subsequently return to baseline following the cessation of stimulation. The magnitude of cytosolic calcium elevation depends on the type, concentration, intensity, and duration of the stimulus, as well as the presence of calcium-buffering proteins (Schwaller, 2010). To maintain calcium homeostasis, cells rely on tightly regulated mechanisms for calcium transport.

Calcium transport in cells is facilitated by channels that permit the Ca^2+^ ion influx, and pumps or exchangers that facilitate Ca^2+^ ion efflux. This process involves sodium-calcium exchangers (NCX), intracellular and plasma membrane calcium channels, and several families of pumps, including Plasma Membrane Calcium ATPase (PMCA), Sarco/Endoplasmic Reticulum Calcium ATPase (SERCA), and Secretory Pathway Calcium ATPase (SPCA) (Cheng et al., 2006). Additional routes of calcium entry comprise TRP channels and ionotropic P2X receptors, which are cation-selective ion channels. Macrophages express channels from multiple TRP (Transient Receptor Potential) subfamilies- TRPC (Canonical), TRPV (Vanilloid), TRPM (Melastatin), TRPML (Mucolipin)- which act as weakly voltage-sensitive, cation-selective channels responding to mechanical, chemical, and thermal stimuli (Desai and Leitinger, 2014).

The calcium channels regulate the transportation of Ca^2+^ ions in response to calcium signaling and cellular demands. Phosphatidylinositol 4,5-bisphosphate (PIP2) and inositol 1,4,5-trisphosphate (IP3) are closely linked molecules that play key roles in calcium signaling inside the cells. PIP2 is hydrolyzed by the enzyme phospholipase C (PLC) into IP3 and diacylglycerol (DAG) upon receptor triggering and activation. Thereafter, IP3 diffuses into the cytoplasm and attaches to its receptor on the endoplasmic reticulum, initiating the release of sequestered Ca^2+^ ions into the cytosol (Rasmussen et al., 1990). The release of ER calcium induces store-operated calcium entry (SOCE) either through calcium release–activated calcium (CRAC) channels, mainly composed of STIM1, i.e., ER Ca^2+^ ion sensor, and ORAI1 (pore-forming subunit), or via Voltage Gated Calcium Channels (VGCC) or both (Vig and Kinet, 2009). This is a fundamental and highly conserved mechanism across all cell types, serving as a versatile intracellular messenger that orchestrates diverse physiological processes (Rasmussen et al., 1990).

Innate immune cells, like macrophages and neutrophils, utilize calcium signals to detect and eliminate pathogens (Chaplin, 2010). Disruptions in calcium homeostasis have been shown to influence disease progression, immune function, and treatment outcomes (Ingale et al., 2025). Macrophages, are elements of the innate immune system, depend on calcium signaling to mediate cytokine production, antigen presentation, and pathogen clearance. The pattern and frequency of Ca^2+^ ion oscillations can influence downstream transcriptional responses such as NF-κB, NFAT, and MAPK activation (Zhou et al., 2006). Inhibition of extracellular Ca^2+^ ion entry in LPS-stimulated macrophages amplified IL-12 production through the CaMKKβ–AMPK–SIRT1 axis, highlighting the sensitivity of immune responses to calcium dynamics (Liu et al., 2016). Likewise, calcium a regulator of the cGAS-STING pathway, which detects cytosolic DNA to trigger interferon production, important for innate immunity and autoimmunity. changes in cytosolic calcium influence STING activation and downstream signaling, including NF-κB and IRF3 transcription factors needed for IFN-γ and inflammatory cytokine expression (Mathavarajah et al., 2019). Distinct calcium signatures are associated with macrophage polarization. Influx of extracellular Ca^2+^ ions has been shown to be required for macrophage polarization toward the pro-inflammatory M1 phenotype (Wu et al., 2023). Elevated mitochondrial Ca^2+^ ion uptake has been reported to enhance anti-inflammatory M2 polarization and augment phagocytic capacity. Conversely, the suppression of the mitochondrial calcium uniporter (MCU) impairs M2 polarization without affecting the M1 phenotype (Tedesco et al., 2019).

### 3.1 Regulation of macrophage effector functions by calcium

Calcium signaling has a direct influence on cytokine production in macrophages. The calcium-sensing receptor (CaSR) is widely expressed in tissues such as monocytes and macrophages. IL-1β and IL-6 upregulate the CaSR and also alter systemic calcium concentrations. These cytokines suppress parathyroid hormone secretion and decrease 1,25-dihydroxyvitamin D levels, which contribute to hypocalcemia in critically ill patients, including those with sepsis. The substantial increase in CaSR expression induced by TNF-α may function as a protective response to inflammation. This mechanism has been demonstrated in murine macrophages, where lipopolysaccharide-induced TNF-α release upregulated CaSR and subsequently inhibited TNF-α synthesis. Altogether, these mechanisms demonstrate the bidirectional relationship between inflammatory processes and calcium homeostasis (Hendy and Canaff, 2016).

Furthermore, antigen binding to immunoreceptors, including the TCR and BCR, the Fcγ and Fcϵ receptors, G protein–coupled chemokine receptors, and some innate pattern-recognition receptors such as Dectin-1, results in the depletion of calcium stores that stimulate store-operated calcium entry through CRAC channels (Feske et al., 2015). In macrophages, studies have demonstrated the involvement of calcium/calmodulin-dependent protein kinase in the pyrimidinoceptor-mediated potentiation of iNOS induction in mouse J774 macrophages (Chen et al., 1998). Research indicates that the rise in [Ca^2+^]_i_ in response to LPS treatment induces ROS generation in murine macrophages (Jin et al., 2006). [Ca^2+^]_i_ governs various functions, including phagocytosis and perhaps phagosome-lysosome fusion.

Phagocytosis, a primary function of macrophages, depends on precise regulation of calcium signaling. Several transcription factors have been identified whose activity is regulated by calcium-activated signaling pathways (West et al., 2001). A notable example is the adenosine 3′,5′-monophosphate (cAMP) response element-binding protein (CREB), which plays important roles in cell survival, proliferation, and differentiation. Its significance in immune function is increasingly recognized. CREB plays an important role in macrophage homeostasis by limiting proinflammatory response and inducing antiapoptotic survival signals in macrophages (Wen et al., 2010). Activation of CREB restricts the intracellular trafficking of *M. tuberculosis* to the phagolysosome by inhibiting MLKL phosphorylation. Therefore, CREB activation constitutes a key mechanism by which *M. tuberculosis* evades the immune response in human macrophages (Leopold Wager et al., 2023).

**Figure 3:**
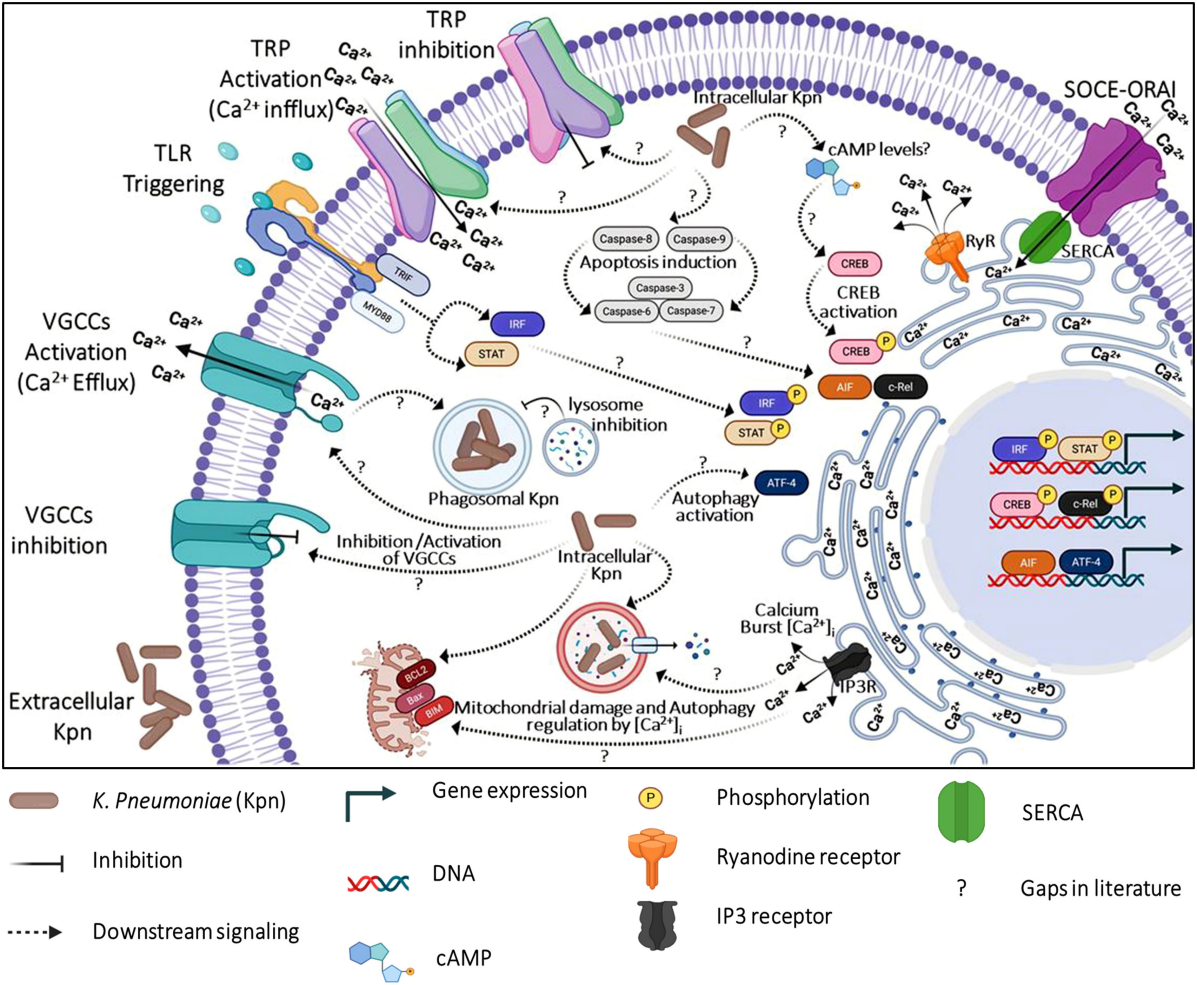
Overview of calcium homeostasis and macrophage effector functions. The figure illustrates the key pathways involved in calcium regulation within macrophages and their impact on effector functions such as phagocytosis, ROS, and pathogen clearance. Intracellular calcium levels are maintained through coordinated activity of plasma membrane channels (e.g., Calcium release-activated calcium channels (CRAC/ORAI1), Transient receptor potential (TRP) channels, Voltage-Gated-Calcium-Channels (VGCCs), endoplasmic reticulum stores (via Inositol triphosphate receptor (IP_3_R), Ryanodine receptor (RyR), and Sarcoplasmic/Endoplasmic Reticulum Calcium ATPase (SERCA), and mitochondrial buffering systems. Multiple mechanisms controlled by calcium homeostasis are still not explored in *K. pneumoniae* infection. Especially, calcium influx/efflux activation and regulation of intracellular calcium levels post-infection. The cross-talk between TLR triggering and calcium-regulated macrophage effector functions is not demonstrated experimentally.

The P2X4 receptor (P2X4R), another endolysosomal ATP-gated calcium channel, contributes to macrophage activation and inflammatory responses. P2X4R expression increases during early phagocytosis in alveolar macrophages, suggesting a role in initiating calcium-driven phagocytic signaling. It modulates IL-1β and IL-18 release via inflammasome activation, linking lysosomal Ca^2+^ ion fluxes to proinflammatory cytokine production (Alharbi and Parrington, 2021). While there have been studies on calcium and calcium channels in macrophages, limited research has been done regarding its role with respect to bacterial infection. Both TRPML and P2X4 Ca^2+^ ion channels are regulators of calcium signaling in macrophage-mediated immune functions. TRPML1-mediated Ca^2+^ release drives phagosome maturation and phagosome-lysosome fusion, processes that engulf and degrade pathogens in macrophages. Knockout of TRPML1 reduces the ability of bone marrow–derived macrophages (BMDMs) to phagocytose large particles, highlighting its requirement for efficient phagocytosis. TRPML2-dependent calcium signaling regulates the release of CCL2/MCP1, promoting macrophage recruitment and migration during inflammation. **Figure 3** gives an overview of calcium homeostasis and reported macrophage effector functions, with persisting gaps in host-pathogen interactions.

### 3.2 Pathogen-mediated disruption of calcium homeostasis

Efficient antigen processing and presentation by macrophages require regulated calcium fluxes. Delvig and Robinson demonstrated that the major histocompatibility complex (MHC) class II-restricted antigen processing in murine macrophages depends on both intracellular [Ca^2+^]_i_ and extracellular Ca^2+^ ion concentration. Their experiments with bacterial epitopes, such as the M5 protein from *Streptococcus pyogenes*, indicated that the calcium fluxes mediated by thapsigargin-sensitive calcium channels and gadolinium-sensitive pathways are crucial for antigen processing. Disruption of intracellular calcium fluxes impaired this process, thereby underscoring the importance of calcium homeostasis in immune activation (Delvig and Robinson, 1999).

*K. pneumoniae* takes advantage of calcium signaling pathways to evade immune responses. In a murine peritonitis model, peritoneal macrophages from wild-type mice exhibited enhanced SOCE via TRPC1 channels following infection. This influx activates M1-specific pathways, including STAT1 and NF-κB p65 phosphorylation, while boosting expression of inflammatory mediators (e.g., NOS2, TNF-α, IL-6, IL-23, CXCL9/10) and maturation markers (MHC-II, CD80/86). Conversely, TRPC1 deficiency impairs these responses, resulting in elevated bacterial burdens in the peritoneum, liver, and blood (Chauhan et al., 2018). In another study of *K. pneumoniae* strains HLJ-D2 and HB-AF5 infection in bovine mammary epithelial cells (bMECs), mitochondrial calcium concentration significantly increased at 1, 3, 6, and 12 hours post-infection when compared to controls contributing to mitochondrial damage and pathogenesis of clinical mastitis (Cheng et al., 2021). However, *K. pneumoniae* facilitates calcium and magnesium homeostasis with DedA family proteins, including YqjA and YghB. These proteins are essential for membrane potential and cation transport, which help in capsule synthesis and resistance to phagocytosis. Therefore, mutant strains that lack these proteins exhibit reduced calcium uptake and increased susceptibility to immune clearance (Tiwari et al., 2024).

Direct causal links between specific Ca^2+^ dynamics and macrophage fate outcomes, such as phagocytosis efficiency, intramacrophage bacterial killing, or apoptosis resistance, are largely absent in *Klebsiella* infection, with existing studies confined to mouse models and lacking human THP-1/PBMC validation, which is needed for clinical translation. Insights into some of these mechanisms can be inferred from studies of other pathogens. For example, *S. aureus* alters calcium fluxes in macrophages, which disrupts cytokine production and inhibits phagosome-lysosome fusion. Inhibiting extracellular calcium influx or intracellular calcium release changes macrophage response, indicating that different calcium sources distinctly regulate immune cell functions (Verma et al., 2020). Similarly, *M. tuberculosis* increases CACNA1S expression, an L-type voltage-gated calcium channel, to manipulate host calcium signaling. This channel enables calcium influx via the MyD88-independent TLR pathway involving pCREB, STIM1, and STIM2. Subsequently, the upregulation of CACNA1S results in suppression of protective innate immune responses, such as phagolysosome fusion and apoptosis promoting immune evasion (Antony et al., 2015). However, the disruption of macrophage calcium homeostasis by *K. pneumoniae* has not yet been experimentally well established. Therefore, investigating the calcium regulation in macrophages upon *K. pneumoniae* infection might open new horizons in the near future to combat the pathogen by developing next-generation therapeutics.

## 4 *K. pneumoniae* pathogenesis and virulence: impact on host immunity

*K. pneumoniae* is a clinically significant opportunistic pathogen that causes life-threatening infections like pneumonia, sepsis, and urinary tract infections, particularly in immunocompromised individuals (Chang et al., 2021). The need to understand its pathogenesis to develop effective treatments is underscored by the rise of multidrug-resistant strains (Ktari et al., 2006). The emergence of hypervirulent (hvKp) is a large hurdle due to their intrinsic capacity to evade immunity and result in deadly infections in healthy individuals (DeLeo et al., 2023). At higher concentrations, hvKp strains exhibit increased capsule and siderophore production, which facilitates immune evasion and promotes the development of invasive diseases such as liver abscesses, meningitis, and endophthalmitis. MDR hvKp strains that combine antibiotic resistance with hypervirulence traits have emerged, posing a serious public health problem. These strains are more frequently reported in clinical settings, often associated with community acquired infection that are resistant and difficult to treat (Dong et al., 2022). *K. pneumoniae* commonly colonizes mucosal surfaces of humans, including the nasopharynx and gastrointestinal tract, with colonization rates varying by site and setting (Martin and Bachman, 2018). Hospitalized patients frequently have increased rates of gastrointestinal colonization, a reservoir for transmission and subsequent infection (Dorman and Short, 2017). Genomic studies have also shown that up to 80% of infections occurring in hospitalized patients come from, colonizing strain, which has a higher incidence (Gorrie et al., 2017). The progression to infection is determined by bacterial density, host risk factors such as immunosuppression, and invasive procedures like endoscopy (Kimmery et al., 1993).

### 4.1 Key virulence factors

#### 4.1.1 Capsular polysaccharide: diversifying the pathogen’s virulence

*K. pneumoniae* establishes infection by defeating host immune defenses and escaping from host tissues through a spectrum of sophisticated virulence factors, such as polysaccharide capsule, lipopolysaccharides, siderophores, and pili (Riwu et al., 2022). The capsule represents the most extensively studied virulence factor of *K. pneumoniae*. It’s a mucoid layer on the bacterial surface, consisting of a repeating glycan polymer that confers protection against host immune responses and facilitates environmental adaptation (Rendueles et al., 2017). There are 79 capsular polysaccharide (CP) serotypes identified in *K. pneumoniae.* Particularly, the capsule serotypes K1 and K2 are recognized as a major virulence factor in classical *K. pneumoniae* (cKP) infections. Similarly, in hypervirulent *K. pneumoniae* (hvKP) strains, eight capsular serotypes have been reported: K1, K2, K5, K16, K20, K54, K57, and the recently identified KN1 (Shon et al., 2013; Lee et al., 2016). **Table 1** summarizes the major hvKP Serotypes and their features (**Table 1**). In addition to aiding colonization and immune evasion, these pathogenic factors also promote severe invasive disease (Catalán-Nájera et al., 2017).

**Table 1:** Major receptors of macrophages and their functions.

Receptor	Ligands recognized	Function in macrophages	Examples of pathogens	References
TLR2	Lipopeptides, peptidoglycan, lipoteichoic acid (LTA), lipoarabinomannan, zymosan, hemagglutinin proteins	Recognizes diverse ligands, promotes uptake of bacteria, viruses, parasites	Bacteria, viruses, mycobacteria, parasites	(Fu and Harrison, 2021; Uematsu and Akira, 2007)
TLR4	LPS on Gram-negative bacteria	Binds LPS, promotes internalization of Gram-negative bacteria	Gram-negative bacteria (e.g., E. coli)	(Uthaisangsook et al., 2002; Fu and Harrison, 2021)
TLR5	Bacterial flagellin	Facilitates the uptake of flagellated bacteria	*P. aeruginosa*	(Fu and Harrison, 2021)
CD14	LPS, co-receptor for TLR4	Co-receptor aiding uptake and clearance of Gram-negative bacteria	NTHi, Acinetobacter baumannii, E. coli	(Knapp et al., 2006)
NLRs	Bacterial peptidoglycan, flagellin, toxins; viral RNA; fungal structures; DAMPs (ATP, uric acid, cholesterol crystals)	Cytosolic sensors recognizing PAMPs/DAMPs, activating immune signaling	Broad microbial and damage signals	(Almeida-da-Silva et al., 2023; Moloudizargari et al., 2019)
RLRs (RIG-I-like receptors)	Viral RNA	Detect RNA viruses and trigger antiviral responses	RNA viruses	(Kawai and Akira, 2009; Yoneyama and Fujita, 2009)

The polysaccharide capsule protects the bacterium from phagocytosis, serum killing, and enhances its ability to survive and infect the host (Struve and Krogfelt, 2003). Studies indicate that the capsule plays a significant role in pathogenesis as encapsulated strains are more resistant to host immune clearance. Genes encoding enzymes and regulatory proteins for polysaccharide production, polymerization, and transport regulate the synthesis of the *K. pneumoniae* capsule from the capsular polysaccharide synthesis (*cps*) locus (Opoku-Temeng et al., 2019). CP inhibits dendritic cell maturation and reduces the dendritic cell-driven production of IL-12, TNF-α, and other cytokines. Consequently, this allows the bacterium to evade host defense and establish infection. CP also suppresses IL-8 expression by interfering with Toll-like receptor (TLR) signaling, especially via the TLR2 and TLR4 pathways (Singh et al., 2025). *K. pneumoniae’*s CP limits the recognition and activation of PRRs by macrophages and neutrophils, leading to innate immune suppression (Lin et al., 2019). Hypermucoviscosity refers to the highly mucoid and viscous phenotype exhibited by *K. pneumoniae* colonies when cultured on agar plates. Infections caused by carbapenem-resistant hypermucoviscous *K. pneumoniae* are associated with high mortality rates. Therefore, it confirms that hypermucoviscosity also serves as a significant virulence factor in *Klebsiella* (Shankar et al., 2018; Kochan et al., 2022).

#### 4.1.2 Lipopolysaccharides: armor against host complement system

Lipopolysaccharides are composed of three distinct components: the lipid-A moiety, a core oligosaccharide, and the O-antigen side chain. The *lpx* gene cluster produces lipid-A, a hydrophobic portion of LPS, which is found in the outer membrane. Lipid-A and the O-antigen are connected via the core oligosaccharide. The O-antigen consists of repeating oligosaccharide units that determine the structural diversity of LPS (Singh et al., 2025). The length of the O-antigen influences bacterial survival; full-length O-antigens (smooth LPS) are less susceptible to complement-mediated killing than truncated O-antigens (rough LPS) (Pennini et al., 2017). *K. pneumoniae* employs LPS to evade host complement-mediated clearance by preventing C3b deposition on the bacterial cell wall (Bulati et al., 2021). Furthermore, the structural modification of the lipid-A component of the pathogen confers resistance to cationic antimicrobial peptides, enhancing the pathogen’s immune evasion and virulence. *K. pneumoniae* LPS interacts with Toll-like receptor 4 (TLR4) on host immune cells and activates NF-κB to induce the production of pro-inflammatory cytokines. This mechanism enables the pathogen to exploit the host immune response to damage host tissues while providing an opportunity to the pathogen to survive and spread further (Regueiro et al., 2009).

#### 4.1.3 Siderophores: the iron importers

**Figure 4:**
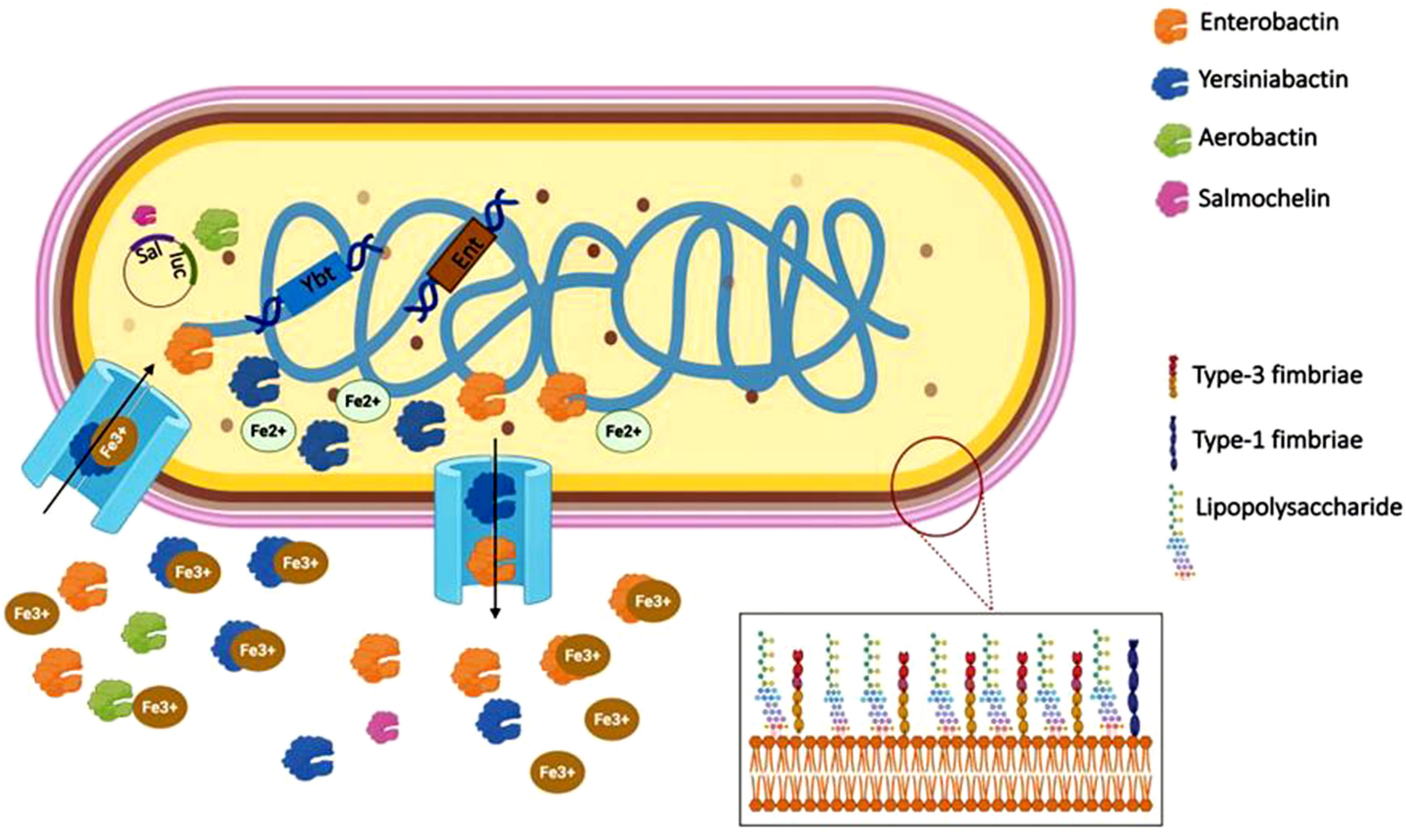
Schematic representation of siderophore-mediated iron acquisition in *Klebsiella pneumoniae*. The bacterial chromosome encodes siderophore biosynthetic gene clusters for Yersiniabactin (Ybt), Aerobactin (Iuc), Salmochelin (Sal) and Enterobactin (Ent). These siderophores are secreted into the extracellular environment where they bind ferric iron (Fe^3+^). The Fe^3+^-siderophore complexes are recognized and transported back into the bacterial cell through specific outer membrane transporters. Once internalized, Fe^3+^ is reduced to Fe^2+^ and released for cellular metabolic processes. The inset depicts the bacterial surface fimbrial structures, including Type 1 fimbriae and Type 3 fimbriae, which contribute to adhesion and biofilm formation.

Iron levels affect not only siderophore production, but also bacterial growth, capsule formation, lipopolysaccharide production, fimbriae expression, and antibacterial activity (Singh et al., 2025). *K. pneumoniae* produces siderophores, small high-affinity iron chelating molecules to counteract iron limitation imposed by the host’s nutritional immunity (Kumar et al., 2024). These include siderophores such as Enterobactin, Aerobactin, Salmochelin, and Yersiniabactin (Lawlor, 2007), which bind to insoluble iron (Fe^3+^) and are subsequently transported into bacterial cells via specialized receptors. This enables the pathogen to secure the nutrient, “Fe,” for survival and virulence. The roles of Enterobactin, Aerobactin, Salmochelin, and Yersiniabactin in iron transport are illustrated in **Figure 4**. In addition to iron acquisition, *K. pneumoniae* manipulates host iron metabolism by inducing transferrin receptor 1 (TFR1) via the STAT6-IL-10 axis. This strategy enhances intracellular iron availability, complementing siderophore activity to support bacterial survival within macrophages (Grubwieser et al., 2023).

#### 4.1.4 Fimbriae: bacterial adhesins

Bacterial surfaces have filamentous, non-flagellar extensions called fimbriae. Type-1, Type-3, and Kpc are the three types of fimbriae that have been found in *K. pneumoniae*. Bacterial cell surfaces have thin, stiff, hair-like features called Type-1 fimbriae. The *fim* gene cluster controls how these structures are formed, and intercellular pathways and chaperone proteins manage their assembly. Type-1 fimbriae expression is controlled by the *fimK* gene, and its absence results in a deficiency of type-1 fimbriae (Riwu et al., 2022). *K. pneumoniae’*s type-1 fimbriae help the bacteria adhere to host cells that have mannose receptors. Moreover, the *mrkABCD* gene cluster, which can be chromosomal or plasmid-derived, spirally paraphrases Type-3 fimbria. Attachment to host cells is mediated by the *fimH* and *mrkD* genes, which separately encode type-1 and type-3 fimbriae (Shah et al., 2017). These fimbrial factors promote colonization, invasion, and pathogenicity, collectively increasing virulence. Chaperone and scaffolding proteins encoded by *mrkB, mrkC*, and *mrkF* genes preserve the stability of fimbrial subunits (Singh et al., 2025).

#### 4.1.5 Secretory systems of bacteria

The secretory systems of *K. pneumoniae* have also been reported as virulence factors in *K. pneumoniae* infections. Hypervirulent *K. pneumoniae* have been shown to have an elevated presence of the Type I Secretion System (T1SS). The diversity of T1SS in *K. pneumoniae* strains indicates the importance of T1SS in infection dynamics. The Type II Secretion System (T2SS) mediates the release of virulence factors, such as the ADP-ribosylating toxin of *K. pneumoniae*. The Type III Secretion System (T3SS), a widely reported pathogenic factor, was not identified in *K. pneumoniae* and remains insufficiently characterized. The Type IV Secretion System (T4SS), commonly found in multidrug-resistant strains, facilitates gene transfer and contributes to antibiotic resistance and virulence. The function of the Type V Secretion System (T5SS) in *K. pneumoniae* remains poorly understood, and its presence is also still unclear (Yang et al., 2023).

Type VI Secretion System (T6SS) is a molecular mechanism used by *K. pneumoniae* to acquire a competitive advantage against the gut microbiota. This long-term colonization of the gut microbiota involves targeting the bacterial competitors (Merciecca et al., 2022). Beyond its role in interbacterial competition and microbiota modulation, T6SS also contributes to immune evasion by delivering effector proteins into host cells, such as the VgrG4 effector, which induces mitochondrial fragmentation and activates immune modulators like NLRX (Liu et al., 2023). This dual function of T6SS in modulating host and microbial interactions underscores its importance in establishing and maintaining *K. pneumoniae* infections (Hsieh et al., 2019). *K. pneumoniae* employs distinct strategies to evade the innate immune response (Codo et al., 2018). *Klebsiella* has become a notorious pathogen that exploits stealth strategies and actively suppresses innate immune defenses to overcome host responses while persisting within tissues (Bengoechea and Sa Pessoa, 2019).

Additional virulence factors in *K. pneumoniae* include efflux pumps, which are involved in functions such as reducing membrane permeability, preventing drug influx, and actively efflux compounds toxic to the pathogens. The presence of the AcrAB-TolC complex in *K. pneumoniae* helps in exporting antibiotics and host-derived antimicrobials. The EefABC efflux pump in *K. pneumoniae* enhances acid and antibiotic resistance, thereby increasing its competitive potential against the host gut microbiota. The RND-type efflux pump, KexD recognizes multiple substrates and functions with AcrA but not with KexA or KexG. The OqxAB, efflux pumps contribute to MDR phenotype in *K. pneumoniae* and can mobilize via plasmid transposition. Notably, the SMR-type efflux pump KpnEF confers resistance to bile, osmotic stress, and promotes capsule synthesis. Likewise, the MFS-type pump KpnGH confers tolerance to antimicrobials and bile salts, and its deficiency causes reduced *K. pneumoniae* growth in hyperosmotic and microaerobic conditions (Borszekova Pulzova et al., 2016).

In summary, the pathogen’s virulence factors modulate protective immunity by disrupting the innate immunity like complement fixation, phagocytosis, etc. Especially, the virulence factors confer an intracellular survival potential by facilitating *K. pneumoniae* to either prevent or remove excess antimicrobial drugs. They further ensure an uninterrupted supply of the limiting factors, for instance, iron and together provide molecular components for firm adhesion to host cells supporting colonization. It is highlighted that reviewing macrophage-specific molecular mechanisms reported to sense microbial invasion and the downstream signaling will help us better understand the host-*K. pneumoniae* interaction.

## 5 Pathogens’ recognition by macrophages

The innate immune system serves as the vanguard of the host defense against microbial pathogens, consisting of physical and anatomical barriers, effector cells, antimicrobial peptides, and other soluble factors (Carroll and Janeway, 1999). Similarly, the innate immune surveillance mechanism within the host confronting microbial pathogens initiates a series of antimicrobial responses determined by the host-pathogen interaction (Kumar et al., 2011). As previously noted, macrophages are required for antigen presentation and various immunoregulatory functions in different pathophysiological circumstances (Chen et al., 2023). In case of bacterial infection, macrophages are vital in the initial immune response by engulfing the bacteria, releasing key cytokines and chemokines that attract neutrophils and other immune cells. On the contrary, the depletion of macrophages enhances the host’s vulnerability to infections, underscoring their role in regulating bacterial proliferation (Bruhn et al., 2015; Qiu et al., 2012).

The detection of microbial pathogens by macrophages is predominantly facilitated by Pattern Recognition Receptors (PRRs), which could be either innate or germline-encoded, identifying PAMPs and Damage-associated Molecular Patterns (DAMPs). The vertebrate innate immune system classifies PRRs into five principal categories based on their protein domain homology: i) Toll-like receptors (TLRs), ii) NLRs, iii) Retinoic acid-inducible gene-I-like Receptors (RLRs), iv) C-type Lectin Receptors (CLRs), and v) Absent in melanoma-2-Like Receptors (ALRs) (Kawai and Akira, 2009; Li and Wu, 2021). The PRRs are composed of ligand recognition domains, intermediate domains, and effector domains, which enable them to bind to microbial markers and recruit adaptor molecules.

One of the major PRRs involved in bacterial infections is the TLRs. Host cells sense various microbial invasions through a diverse set of Toll-like receptors, each tuned to specific pathogen-associated molecules. *K. pneumoniae* activates diverse PRRs in macrophages to modulate immune responses and promote its survival. Among Toll-like receptors, TLR4 recognizes *Klebsiella’s* lipid A (LPS), capsular polysaccharides (CPS), murein lipoprotein (LppA), and acylpolygalactosyl (APG), TLR2 detects OmpA. TLR3 senses host-derived dsRNA and TLR9 recognizes unmethylated CpG motifs in bacterial DNA (Li et al., 2025). Multiple signaling cascades are activated as a result of the above interaction between the specific receptors and their ligands (Mogensen, 2009). **Table 2** summarizes the major receptors found on macrophages, their ligands, and their functions (**Table 2**).

**Table 2:** Major hvKP Serotypes and their features.

Serotype	Clinical association	Virulence traits	References
K1 (KL1)	Liver abscess, sepsis	Highest virulence, sialic acid mimicry	(Ko, 2017)
K2 (KL2)	Meningitis, lung abscess	Strong phagocytosis resistance, high serum survival	(Ko, 2017)
K5	Highly virulent capsular type	Intra- and extra-pulmonary infections	(Jiang et al., 2024)
K20	Liver abscess	Serum survival	(Lin et al., 2017)
K54	Liver abscess, meningitis	Moderate virulence	(Xu et al., 2024)
K57	Pyogenic liver abscesses	Adhesion and serum resistance	(Xu et al., 2024)

Multiple studies have emphasized the importance of these receptors in the pathogenesis of human diseases. Mutations in human TLR4 have shown a higher incidence of Gram-negative infections in already immunocompromised patients compared to wild type (Agnese et al., 2002). Similarly, in sepsis patients with a single nucleotide polymorphism in their TLR1 gene display a hypermorphic effect leading to increased susceptibility to organ dysfunction and death in comparison to patients with a normal genotype (Wurfel et al., 2008). Yet another separate meta-analysis study revealed that TLR2-mutated individuals are at a greater risk of tuberculosis development (Hu et al., 2019). A significantly higher bacterial burden was observed in *Salmonella* colonization of TLR2/4 double-knockout mice, when compared to the wild-type mice (Weiss et al., 2004). Yet another study has identified that the 3d mice lacking endosomal TLR signaling due to UNC93B1 mutation, which prevented proper localization of endosomal TLRs, causing high susceptibility to *Klebsiella* even at sublethal doses (De Gaetano et al., 2025).

### 5.1 Pathogen post-recognition and downstream signaling

Upon engagement of TLRs, either of the two signaling pathways-the MyD88-dependent and TRIF-dependent- associates with a characteristic of TLR2 and TLR4 triggering by microbial pathogens. The TLR2/4 triggering recruits MyD88, and associates with IRAK4/IRAK1 (Deguine and Barton, 2014; Pereira et al., 2022), subsequently activating TRAF6 (Pereira et al., 2022). This initiates downstream activation of TAK1, resulting in phosphorylation of MAPKs and activation of NF-κB (Deguine and Barton, 2014). Finally, promoting the transcription of proinflammatory cytokines like IL-1, IL-6, and TNF-α. On the contrary, the TRIF-dependent pathway, activated primarily by TLR3 and TLR4, recruits TRIF, which interacts with TRAF3 and ultimately activates IRF3, leading to the production of type I interferons (IFNs) (Hu et al., 2015). The integration of MyD88 and TRIF pathways, particularly in TLR4 signaling, enables a more comprehensive immune response against diverse pathogens (Pereira et al., 2022).

**Figure 5:**
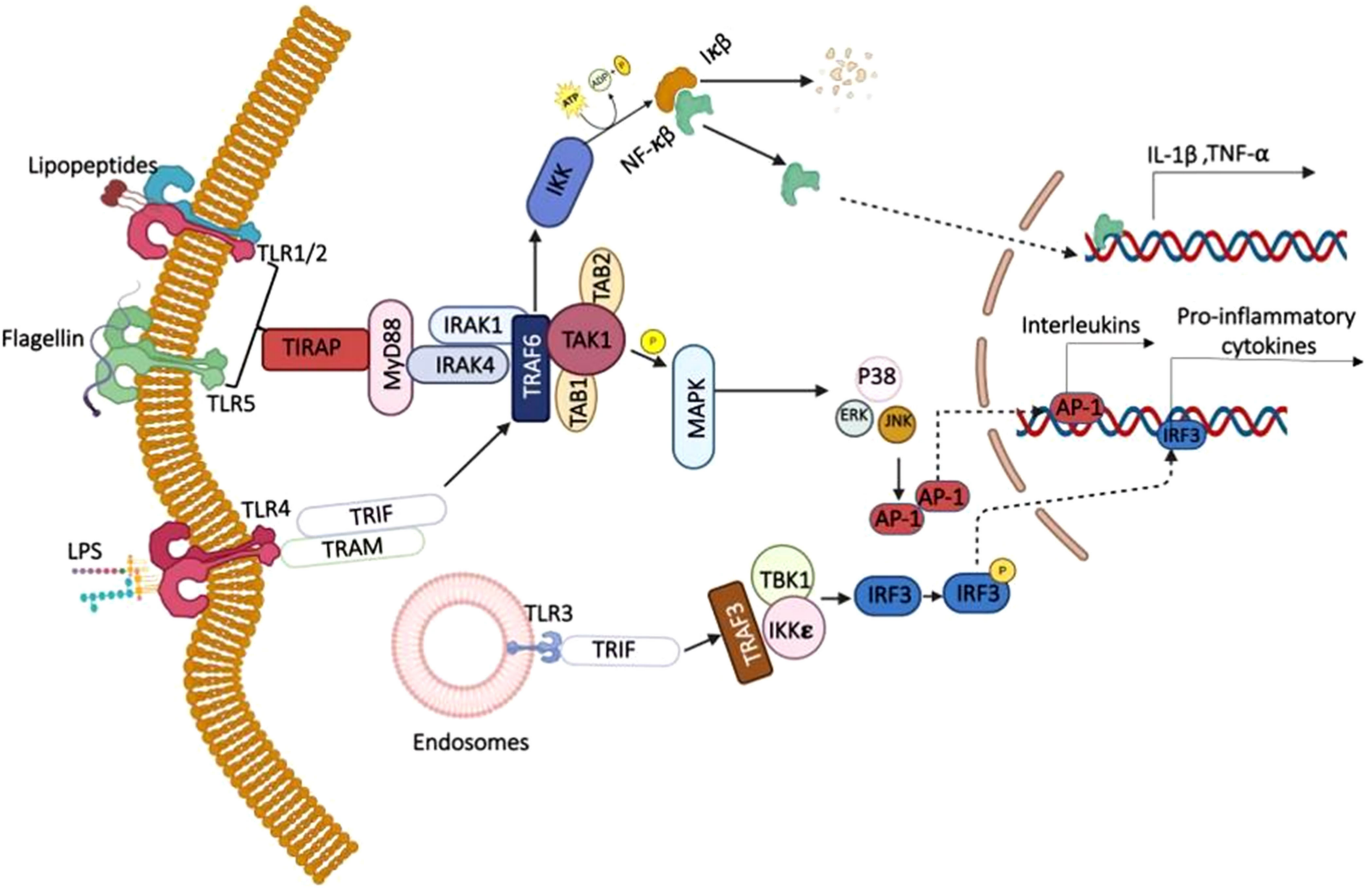
Recognition of pathogen-associated ligands by TLRs. TLR4,5,1/2 activates MyD88-dependent pathway. In the MyD88-dependent pathway, TIRAP, an adaptor protein, helps transmit signals from TLR4 to MyD88, which then recruits IRAK4 and TRAF6. TRAF6 activates the TAK1–TAB1/2 complex, leading to phosphorylation of the IKK complex as well as MAP kinases. The former results in IκB degradation, allowing NF-κB to translocate into the nucleus, whereas the latter results in the phosphorylation and activation of AP-1. Together, NF-κB and AP-1 induce the production of proinflammatory cytokines. In the TRIF-dependent pathway, TLR4 in the late phase recruits TRAM and TRIF, which interact with TRAF6 and converge on the same downstream MyD88 pathway. Endosomal TLR3 signals solely via TRIF, triggering the TBK1/IKK ϵ–IRF3 that drives inflammatory gene expression.

The MyD88 pathway usually activates NF-κB instantly but only for a short while, whereas the TRIF pathway activates NF-κB and IRF3 more slowly and for longer, leading to the production of both inflammatory cytokines and Type I IFN (Hu et al., 2015). Certainly, the activation of two pathways induces the production of inflammatory cytokines and fights against microbial pathogens, as depicted in **Figure 5**. Likewise, NLRP3 and NLRC3 of the NLR family of receptors influence inflammatory responses of macrophages through the development of the inflammasome. NLRP3 activation leads to the formation of the NLRP3 inflammasome, which has ASC and caspase-1 within it, which in turn activate IL-1β and IL-18 (Ding et al., 2024; Shao et al., 2015; Yao et al., 2024). This pathway triggers pyroptosis, a type of cell death that causes inflammation and helps eliminate pathogens by destroying their niche (Shao et al., 2015). Conversely, NLRC3 acts as a negative regulator by inhibiting TRAF6-mediated NF-κB activation and inflammasome assembly, thus preserving macrophage homeostasis (Eren et al., 2017; Shao et al., 2015).

The functional responses to the signaling in macrophages are disparate and diverse. The DAMPs and PAMPs, when recognized by the PRRs, lead to the production of antimicrobial peptides (AMPs) like defensins and cathelicidins (Basu et al., 2012). This disrupts the integrity of microbial membranes, thereby eliminates the pathogens. TLR stimulation induces the generation of Reactive Oxygen Species (ROS) and Reactive Nitrogen Species (RNS) through NADPH oxidase and Inducible nitric oxide synthase (iNOS), which have significant efficacy against numerous bacterial species (Canton et al., 2021; Wang et al., 2019). The formation of mitochondrial ROS, enabled by the activation of the TRAF6-ECSIT complex, enhances antimicrobial defenses and functions as a secondary messenger in cytokine signaling (Silwal et al., 2020). The TLR signaling cascade also promotes the release of chemokines and proinflammatory cytokines like TNF-α, IL-6, and IL-1β to the site of infection for effective pathogen clearance. For instance, upon recognizing mycobacteria, TLRs prompt macrophages to produce IL-12 and TNF-alpha. TNF-alpha production during MTB infection has been primarily due to TLR2-triggering (Underhill et al., 1999; Flynn and Chan, 2001).

Furthermore, CXCL1 and CCL2 two important cytokines, attract neutrophils and monocytes to the site of infection for an effective immune response against the invading pathogens (T. Liu et al., 2017; Boro and Balaji, 2017). Actively withholding the nutrients is an alternative mechanism employed by macrophages to effectively eliminate the invading pathogens. This process is termed nutrient sequestration, also known as nutritional immunity. Restricting the availability of iron for bacterial growth and virulence, is one such mechanism. Similarly, manganese sequestration has been achieved through the action of natural resistance-associated macrophage protein 1 (NRAMP1), which pumps manganese out of the phagosome (Kehl-Fie and Skaar, 2010; Wessling-Resnick, 2015). In conclusion, though macrophages possess multiple mechanisms to eliminate pathogens, many intracellular pathogens have evolved sophisticated immune evasion strategies that inhibit macrophage cell death, thereby sustaining the macrophages as a niche for their survival and replication. Therefore, a complete understanding of macrophage cell death and survival mechanisms upon bacterial infection appears to be more essential.

## 6 Orchestration of cell death pathways upon *K. pneumoniae* infection

Programmed cell death is a regulated process mediated by specific molecular machinery for maintaining health, and it also contributes to the pathogenesis of various diseases. A crucial aspect of the immune responses during bacterial infections is the coordinated regulation of host cell death pathways, comprising apoptosis, necroptosis, and pyroptosis (Wanford et al., 2022). The cell death pathways fall into two main classifications: i) Accidental Cell Death (ACD) and ii) Regulated Cell Death (RCD) (Lee et al., 2023). ACD happens because of major catastrophic events, while RCD involves programmed cell death (Galluzzi et al., 2015). RCD types include apoptosis, autophagy, necrosis, lysosome-dependent cell death, and pyroptosis; all of which are sites of morphological and biochemical changes, although some of them may have some similarities in mechanism (Codo et al., 2018).

### 6.1 Inflammatory cell death

Necrosis, an unregulated form of cell death, occurs due to acute injury. Numerous changes occur during necrosis, such as cellular swelling, membrane rupture, and discharge of intracellular contents, which activate an immune response (Tonnus et al., 2019). In contrast, necrosis is not caspase-mediated; rather, it is frequently reported in instances involving inflammation beyond the boundaries of the tissue area. *K. pneumoniae* causes necrosis of HepG2 cells through ROS generation, membrane rupture, and secretion of endogenous ligands, i.e., DAMPs, that exacerbate tissue damage. By destabilizing cellular integrity and promoting lysosomal enzyme release, *K. pneumoniae* amplifies inflammation, disrupts hepatocyte function, and facilitates bacterial spread (Yang et al., 2012).

Pyroptosis is an inflammatory form of RCD that occurs due to infection or cellular stress, which leads to the formation of pores in the plasma membrane by proteins known as gasdermins (Yu et al., 2021). It is characterized by the production of pro-inflammatory cytokines, such as IL-1β and IL-18, that boost immunity. *K. pneumoniae* activates the NLRP3 and NLRC4 inflammasomes in bone-marrow-derived macrophage cells, leading to caspase-1 activation and IL-1β production. *K. pneumoniae* strains like A28006 induce robust pyroptosis, releasing lactate dehydrogenase (LDH) and pro-inflammatory mediators that promote bacterial clearance. In contrast, the A54970 strain of *K. pneumoniae* suppresses inflammasome activation and pyroptosis by enhancing IL-10 production, which inhibits caspase-1 activity and IL-1β secretion, facilitating bacterial survival and dissemination. Consequently, pyroptosis raises the bacteria’s vulnerability to microbicidal agents such as hydrogen peroxide, underscoring its protective role in host defense (Codo et al., 2018).

### 6.2 Non-inflammatory cell death

Apoptosis is a programmed form of cell death for maintaining tissue homeostasis and involves the systematic degradation of cellular components through caspase activation. It is characterized by cell shrinkage, chromatin condensation, and apoptotic body formation, occurring through intrinsic or extrinsic pathways. *Neisseria gonorrhoeae* induces macrophage apoptosis primarily via the mitochondrial pathway, modulating pro-apoptotic and anti-apoptotic factors such as Bax and Bcl-xL (Dell’Annunziata et al., 2024). Additionally, *K. pneumoniae*-derived Outer membrane vesicles (OMVs) strengthen this pro-apoptotic effect by increasing the expression of Bax and Bim, while decreasing Bcl-xL, as well as disrupting the mitochondrial membrane and releasing cytochrome c during infection of HepG2 cells. The entire process involves the activation of Apaf-1, followed by the activation of caspase-9. However, *K. pneumoniae* has the ability to delay apoptosis of immune cells like neutrophils by modulating the Bax/Bcl-2 ratio and increasing Mcl-1 expression, which inhibits caspase-3 activation. Therefore, the pathogen exists inside cells over an extended period of time, avoiding immune defense mechanisms, whereas exacerbation of apoptosis has been reported under certain circumstances, such as with hypervirulent strains. Thus, *K. pneumoniae* inhibits efferocytosis, thereby escalating secondary necrosis, inflammation, and bacterial dissemination (Yang et al., 2012).

Autophagy is a major cellular pathway that is fundamentally based on the degradation of cytosolic components packed into autophagosomes, which in turn fuse with lysosomes for their degradation. Autophagy activation by the PI3K-AKT-mTOR pathway is enabled by bacterial degradation during *K. pneumoniae* infection of *Caenorhabditis elegans* (Kamaladevi and Balamurugan, 2017). Although the pathogen subverts this process by recruiting Rab14 to block phagosome maturation and Atg7 to prevent inflammation. These strategies are well documented, but the molecular details of how *K. pneumoniae* exploits host autophagy machinery remain incomplete. However, not much is known about how specific virulence factors and host receptors regulate autophagy modulation during infection. Furthermore, there is an important need to connect autophagy with other immune responses, such as cytokine production and cell death. These gaps confirm the need for further detailed investigation studies to unravel the manipulation of autophagy by *K. pneumoniae* (Wei et al., 2023). Autophagy is a mechanism used by macrophage for resistance against *K. pneumoniae* infection. Research shows that *K. pneumoniae* can activate ATG7, which is in turn involved in autophagosomal membrane elongation, triggering autophagy. The *atg7* KO mice are more susceptible to *K. pneumoniae* infection, have reduced bacterial clearance, enhanced lung injury, and decreased survival rates. The mechanism involves ATG7 competitively inhibiting the interaction between p-IκBα and ubiquitin, reducing p-IκBα ubiquitination, which promotes NF-κB translocation to the nucleus, and increasing inflammatory damage (Li et al., 2025).

Ferroptosis is a distinct form of iron-dependent regulated cell death driven by the accumulation of lipid peroxides to lethal levels within the cells (Dixon et al., 2012). This phenomenon has been associated with disrupted iron homeostasis, oxidative stress due to increased ROS, and suppression of antioxidant status, especially through glutathione peroxidase 4 (GPX4) (Xie et al., 2016). As an emerging area of study, ferroptosis has been investigated for its role in various diseases, offering potential therapeutic avenues. In bacterial and viral infections, ferroptosis displays diverse interactions. For example, an opportunistic pathogen, *Pseudomonas aeruginosa* causes ferroptosis in bronchial epithelial cells through the production of 15-hydroperoxy-AA-PE via its lipoxygenase enzyme, pLoxA (Sadikot et al., 2005). The RNS produced by host macrophages can, in turn, neutralize this process and inhibit phospholipid peroxidation.

*M. tuberculosis* infection induces ferroptosis in host macrophages by decreasing GPX4 levels and increasing lipid peroxidation, ROS, and free iron concentrations. Although iron chelation and ferroptosis inhibitors such as ferrostatin-1 can mitigate these effects, *Mycobacterium* employs secreted proteins to neutralize ROS, thereby complicating its interaction with ferroptosis pathways (Amaral et al., 2019). In viral infections, particularly those caused by SARS-CoV-2, ferroptosis is an important factor in disease progression (Xia et al., 2021). SARS-CoV-2 disrupts iron metabolism, resulting in iron overload and excessive ROS production, while also suppressing GPX4 expression. These alterations accelerate lipid peroxidation and cell death. Therapeutic strategies, including selenium supplementation and iron chelation, have demonstrated potential to reduce the damage associated with SARS-CoV-2-induced ferroptosis (Wang et al., 2021).

**Figure 6:**
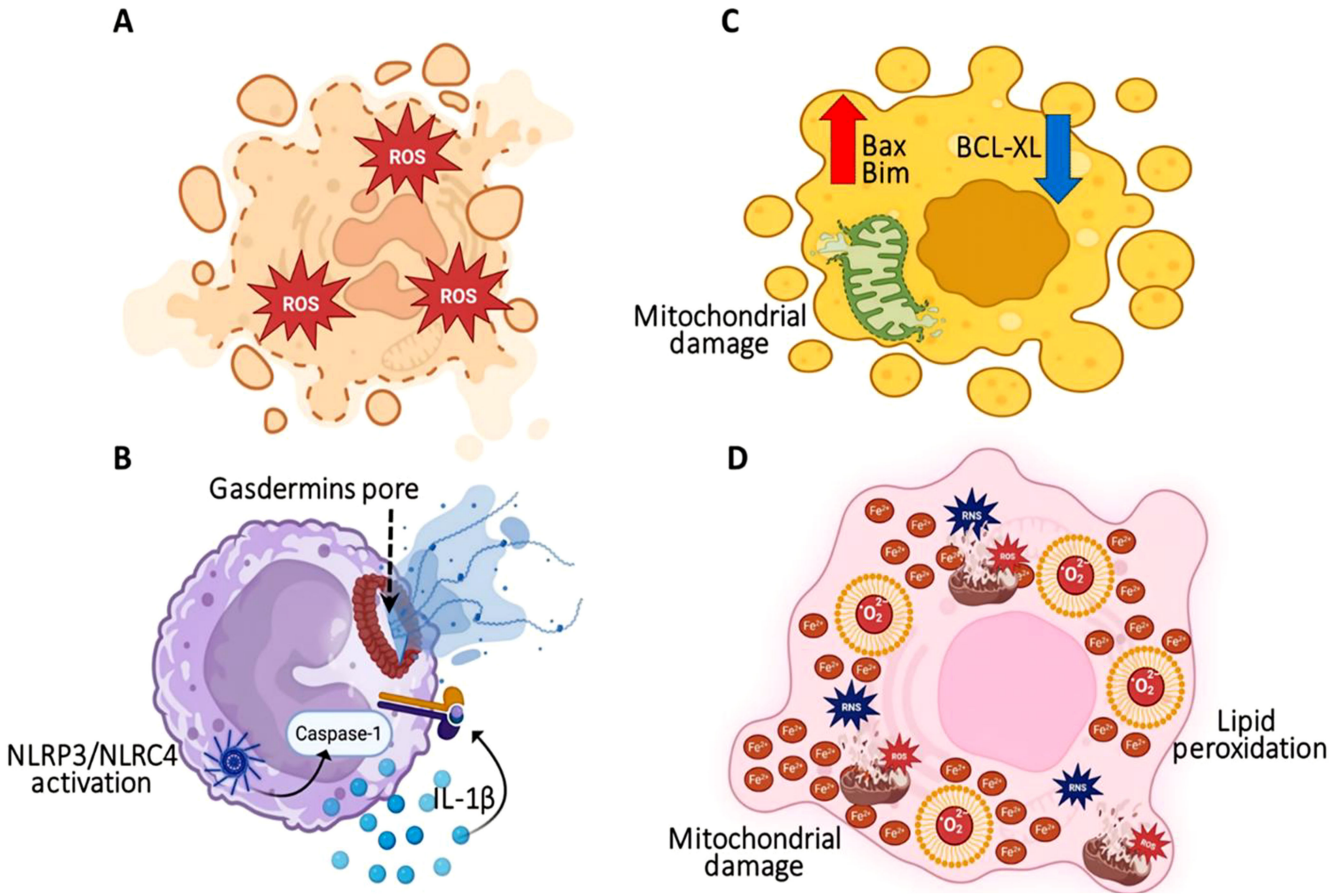
Induction of inflammatory and non-inflammatory cell death. **(A)** Necrosis, an unregulated form of cell death. Excessive generation of ROS levels promotes oxidative membrane damage and loss of cellular integrity, leading to necrosis. **(B)** Pyroptosis is an inflammatory form of RCD characterized by the production of pro-inflammatory cytokines, such as IL-1β and IL-18, and occurs due to infection or cellular stress, which results in the formation of a pore in the plasma membrane by gasdermin. *K*. *pneumoniae* activates the NLRP3 and NLRC4 inflammasomes in Bone-marrow-derived macrophage cells, leading to caspase-1 activation and IL-1β production. **(C)** Induction of macrophage apoptosis by modulating pro-apoptotic and anti-apoptotic factors such as BAX and Bcl-xL via mitochondrial disruption, leading to the release of cytochrome c. **(D)** Ferroptosis is a distinct form of iron-dependent regulated cell death driven by the accumulated ROS, leading to the oxidation of polyunsaturated fatty acids in the plasma membrane.

The involvement of ferroptosis in *K. pneumoniae* infections appears to vary by cell type. In bovine mammary epithelial cells, infection with *K. pneumoniae* triggers classical ferroptosis, characterized by iron accumulation, increased lipid peroxidation, enhanced ROS levels, and a suppression of the Nrf2/xCT/GPX4 antioxidant pathway, ultimately resulting in epithelial injury and inflammatory responses. The use of ferrostatin-1 to inhibit ferroptosis restores antioxidant signaling and reduces inflammation, emphasizing ferroptosis as a major contributor to tissue damage during mastitis (Mao et al., 2025). Conversely, in a mouse model of pulmonary infection, carbapenem-resistant hypervirulent *K. pneumoniae* inhibits ferroptosis-related pathways in macrophages by upregulating SLC7A11 and glutathione, which restricts iron-dependent oxidative stress and promotes the survival of bacteria within cells. Interestingly, reactivation of lipid peroxidation related to ferroptosis in macrophages improves bacterial clearance without causing significant macrophage death (Yu et al., 2025). Recent macrophage infection models further support this distinction by demonstrating modulation of iron-dependent oxidative stress pathways. These include alterations in glutathione metabolism, lipid peroxidation, and ferroptosis-associated regulators such as SLC7A11. Such findings suggest the presence of ferroptosis-associated redox signaling rather than the execution of classical ferroptotic cell death. Collectively, these results underscore a context-dependent role for ferroptosis in *K. pneumoniae* pathogenesis. Epithelial ferroptosis appears to contribute to tissue injury, whereas suppression of ferroptosis-associated oxidative mechanisms in macrophages promotes immune evasion and intracellular persistence. **Figure 6** summarizes the inflammatory and non-inflammatory forms of cell death. Although apoptosis, autophagy, necrosis, and pyroptosis are well-established contributors to macrophage responses during *K. pneumoniae* infection, the precise contribution of ferroptosis in immune cells remains incompletely understood and requires further investigation.

## 7 Autophagy as a survival strategy in infected macrophages

Autophagy is a highly conserved process through which eukaryotic cells sequester their own cytoplasmic contents into a phagophore and later recycle them via lysosomes for the metabolic processes of the cell (Gomes and Dikic, 2014). Autophagy is classified into three types, namely Macroautophagy, microautophagy, and chaperone-mediated autophagy (CMP) (Parzych and Klionsky, 2014). Macroautophagy makes use of autophagosomes to traffic the extracellular components into the cell, which subsequently fuses with lysosomes for degradation. The cytosolic components are directly taken up by the lysosomes via membrane invagination in microautophagy, whereas chaperons transport the targeted proteins across the lysosomal membrane for their degradation in CMP (Glick et al., 2010).

### 7.1 Autophagic mechanisms in pathogen elimination

The host cells utilize the mechanism of autophagy for their survival upon infection and manage pathogen elimination, regulate inflammation, and maintain cellular homeostasis (Bah and Vergne, 2017). This mechanism allows macrophages to eliminate intracellular pathogens by a process called xenophagy (Gomes and Dikic, 2014), or discard damaged organelles via mitophagy (Youle et al., 2014), and further modulate immune signaling pathways. Autophagy has been triggered by multiple events that stimulate innate immune signaling to fulfill dual protective functions: eliminating inflammatory causes and curtailing excessive immune activation. The above interaction facilitates efficient pathogen elimination while reducing uncontrolled inflammation (Chauhan et al., 2022). Thus, the interplay between autophagy and inflammation is crucial for maintaining cellular homeostasis during infection and innate immune activation.

#### 7.1.1 Xenophagy: discriminatory elimination of intracellular pathogens

Xenophagy is a form of selective autophagy that targets invading microbes for lysosomal degradation. The pathogens that enter host cells initially reside in a pathogen-containing vacuole (Chaudhary and Miller, 2019). When these vacuoles rupture, bacterial components are exposed in the cytoplasm, which are then tagged with ubiquitin molecules. Consequently, the tagged molecules are recognized by autophagy receptors, which then fuse with lysosomes, degrading the bacterial components (Levine, 2005). In *Salmonella typhimurium*, galectins identify compromised phagosomal membranes and activate ubiquitin ligases, thereby initiating xenophagy in endothelial cells (Birmingham et al., 2006). In macrophages, during wild-type *M. tuberculosis* infection, the bacterial ESX-1 system tags bacterial components through ubiquitination and subsequently delivers them to autophagosomes (Watson et al., 2012). In addition to conventional autophagy, macrophages utilize LC3-associated phagocytosis (LAP), a non-canonical mechanism that directly conjugates LC3 to single membrane phagosome-forming structures called LAPosomes, which then mature into phagolysosomes. LAP relies on Rubicon, Beclin-1, and ROS generated from NADPH oxidase 2 (NOX2) for its initiation (Gluschko et al., 2018; Herb et al., 2020). The intracellular pathogen *Listeria monocytogenes* has been reported to be killed by LAP in macrophages rather than the canonical autophagy pathway (Gluschko et al., 2018).

### 7.2 Autophagy in the regulation of inflammation

Autophagy alleviates inflammasomal activation by eliminating damaged mitochondria through mitophagy (Wang S. et al., 2023). Dysfunctional mitochondria emit ROS and disperse their mitochondrial DNA (mtDNA), which are powerful activators of the NLRP3 inflammasome. NLRP3 inflammasome has been reported for its host defense against multiple bacterial invasions (Kelley et al., 2019). In *P. aeruginosa* infections, the deficiency of Atg7 in macrophages leads to the buildup of mtDNA and increased release of IL-1β, inducing pyroptosis (Pu et al., 2017). Furthermore, autophagy directly degrades inflammasome components through proteins like TRIM20 and Immunity-Related GTPase family M (IRGM). These proteins interact with inflammasome subunits, such as NLRP3 and AIM2, inducing autophagic destruction (Traver et al., 2011). Additionally, autophagy governs immune responses by influencing cytokine production and macrophage polarization (Wu and Lu, 2019). M2 macrophages lacking Atg5 exhibit a proinflammatory transformation to M1-like phenotype characterized by elevated production of TNF-α and IL-6. These findings highlight autophagy as a pivotal regulator in modulating pro- and anti-inflammatory macrophage states.

### 7.3 Pathogen tactics to undermine autophagy

It is imperative that pathogens develop novel strategies that sabotage host surveillance and elimination mechanisms. The intracellular defense mechanisms, like production of ROS, RNS, phagosome-lysosome fusion and degradation pathways, must be evaded successfully for intracellular survival (Colombo, 2005). To circumvent xenophagy, pathogens have developed strategies to avoid their detection. For example, *L. monocytogenes* synthesizes ActA and InlK proteins that attract host actin and major vault protein and thus camouflages under the host proteins and thereby inhibits detection by autophagy receptors (Dortet et al., 2012). *Francisella tularensis* synthesizes O-antigen polysaccharides that protect the bacterium from cytosolic sensors, thus evading autophagic targeting (Jessop et al., 2025). Pathogens can suppress autophagy induction as seen in *S. typhimurium* infection. The bacteria stimulate the mTOR pathway through FAK/AKT signaling and degrade AMPK, hence inhibiting autophagy (Ganesan et al., 2017). Likewise, *M. tuberculosis* employs host miRNA miR-30c-1-3p to suppress the production of ATG4B and ATG9B, thereby obstructing autophagosome biogenesis (Peng et al., 2025). Some pathogens utilize the autophagic route as a site for replication. *Brucella melitensis* stimulates the production of autophagosomes to create Brucella-Containing Vacuoles (BCVs), facilitating bacterial multiplication (Qin et al., 2024). Similarly, *Coxiella burnetii* utilizes the effector protein Cig2 to facilitate the fusion of autophagosomes with its vacuole, thereby enlarging the replicative compartment and enhancing bacterial viability (Kohler et al., 2016).

Macrophages employ diverse cell death pathways during *K. pneumoniae* infection, forming a unified model where autophagy serves as the primary defense mechanism, while *K. pneumoniae* strategically shifts the balance toward inflammatory lysis (pyroptosis/necroptosis) and away from immunologically silent apoptosis to evade clearance, thereby amplifying pathogenesis. Autophagy, driven by ATG7 activation, promotes KP degradation via autophagolysosome formation, but *K. pneumoniae* evades this by PI3K–AKT–mTOR/Rab14-mediated xenophagy, allowing intracellular replication. *K. pneumoniae* preferentially induces pyroptosis (NLRP3/NLRC4/caspase-1/11–GSDMD) and necroptosis, which release IL-1β/IL-18 and DAMPs to recruit neutrophils while compromising membrane integrity for bacterial escape; deficiencies in these pathways (NLRP3, caspase-11, NLRC4, IL-1R1) increase mortality by impairing early phagocytic killing, highlighting their net protective role despite pathology. Conversely, *K. pneumoniae* suppresses apoptosis via CPS-mediated Bax/Bcl-2 modulation and Mcl-1 upregulation (primarily in neutrophils, with macrophage implications), preventing PS exposure and efferocytosis to favor dissemination (Li et al., 2025).

### 7.4 Implications of autophagy dysregulation

Genetic and functional defects in autophagy compromise the macrophages’ capacity to eliminate intracellular infections. Studies have shown that conditional knockout of Atg5 in myeloid cell lineages resulted in increased *M. tuberculosis* burden. Similarly, the deficiency of Atg7 in alveolar macrophages worsens *P. aeruginosa* sepsis due to impaired mitophagy (Wang et al., 2025; Pu et al., 2017). The absence of autophagy leads to unregulated inflammasome activation and increased inflammatory damage. Ultimately, a defect in autophagy leads to increased metabolic depletion of macrophages. Notably, the accumulation of impaired mitochondria and misfolded protein aggregates depletes energy reserves, which further impair phagocytic function and reduce cellular viability upon infection or food deprivation (Tabibzadeh, 2023). Autophagy in macrophages functions at the intersection of antimicrobial defense, inflammatory management, and cellular homeostasis and is involved in eradicating intracellular infections. However, it is often exploited and undermined by many pathogens (Colombo, 2005; Peng et al., 2025; Qin et al., 2024; Kohler et al., 2016).

Intracellular pathogens prevent macrophage death to promote their replication. For example, the obligate intracellular pathogen *C. burnetii* releases effectors that prevent mitochondria-dependent apoptosis and also inhibit pyroptosis through IcaA-mediated suppression of caspase-11. It is also reported that NLRP3/caspase-1activation is observed due to cytosolic LPS exposure. On the contrary, Legionella pneumophila utilizes its T4SS for prolonged activation of NF-κB signaling to increase pro-survival factors such as BCL-2 and SidF. Likewise, *Leishmania* subverts macrophage apoptosis by activating the host PI3K/AKT pathways, which leads to the phosphorylation of BAD, further promoting cell survival (Chow et al., 2016). However, pathogens have developed intricate strategies to counteract autophagy, leading to a dynamic interaction that influences infection outcomes. Unraveling how pathogens exploit macrophage survival to ensure their own persistence promises novel avenues.

## 8 Impact of *K. pneumoniae* infection on macrophage defense mechanisms

Besides having a primary role in identification and eliminating pathogens, macrophages have also served as a bridge between innate and adaptive immunity. Therefore, it’s no surprise that human pathogens like K. pneumoniae have evolved sophisticated strategies that manipulate macrophage defense mechanisms in their favor, enabling survival and replication within the host. In particular, macrophages infected with *K. pneumoniae* exhibit increased glycolysis and activation of the IRG1–itaconate anti-inflammatory pathway, a hallmark of rearrangement of the TCA cycle (Li et al., 2025). *K. pneumoniae* can adapt and exploit the microenvironment of the host. This adaptability is the reason behind its pathogenic success. The bacterium alters macrophage responses by inducing an IFN-mediated signaling cascade that promotes an anti-inflammatory macrophage phenotype. This phenotype is defined by increased production of IL-10, which suppresses AIM2 inflammasome activation and reduces secretion of IL-1β, thereby facilitating bacterial persistence.

**Figure 7:**
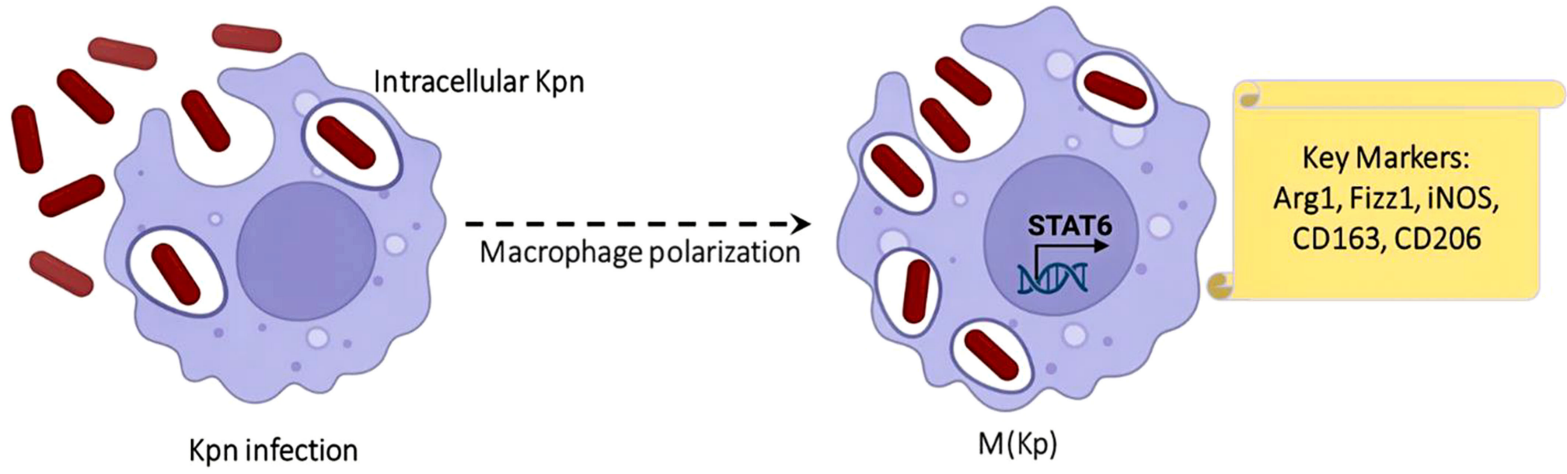
Macrophage polarization upon *K. pneumoniae* infection. *K. pneumoniae* induces a polarization state, M(Kp), resembling M2 macrophages. This state is mediated by STAT6 signaling and characterized by upregulation of Arg1, Fizz1, iNOS, CD163, and CD206.

Furthermore, *K. pneumoniae* manipulates the Toll/interleukin-1 receptor domain-containing adaptor molecule 1 (TICAM-1) to suppress MyD88- and TRIF-mediated inflammatory pathways. This downregulates the activation of key mitogen-activated protein (MAP) kinases, including extracellular signal-regulated kinase (ERK) and c-Jun N-terminal kinase (JNK), which impairs robust immune responses (Feriotti et al., 2022; Kim et al., 2021). The STAT6-IL-10 axis is also important in the aspect of immune modulation by *K. pneumoniae*. Transferrin receptor 1 (TFR1) expression in macrophages is induced by the activation of this pathway, which in turn increases iron uptake and promotes intracellular survival of bacteria. Inhibition of IL-10 or STAT6 markedly reduces TFR1 expression, limiting iron availability and decreasing its survival (Grubwieser et al., 2023). A novel macrophage polarization state, termed “M(Kp),” has been identified by recent studies during infection with *K. pneumoniae*. This anti-inflammatory M2-like polarization state differs from any of the known M2 subtypes and is driven explicitly by activation of the TLR2/4-type I IFN–IL-10–STAT6 signaling axis (Dumigan et al., 2022). The key markers in M(Kp) polarization include Fizz1, Arg1, iNOS, CD163, CD206, and type I IFN and IL-10 signaling-regulated genes, along with a reduction of MHC-II and iNOS expression (Li et al., 2025) as depicted in **Figure 7**.

Disruption of anatomical barriers is a cornerstone of the *Klebsiella* pathogenesis. Chronic obstructive pulmonary disease (COPD), smoking, and mechanical ventilation weaken the epithelial integrity of the respiratory tract. This facilitates bacterial adherence and invasion (Hakansson et al., 2018). CP and fimbriae help in adherence to epithelial cells and resistance to immune clearance (Asokan et al., 2025). In the gastrointestinal tract, *K. pneumoniae* is normally a commensal organism. However, under conditions such as antibiotic-induced dysbiosis, it can transition to a pathogenic state (Joseph et al., 2021). Dysbiosis allows *K. pneumoniae* to dominate the gut microbiome, adhere to the intestinal lining, and translocate to sterile sites, resulting in systemic infections. Hypervirulent strains intensify this process via enhanced adhesive and immune-evasive properties (Hsu et al., 2015). Antibiotic pre-treatment can trigger dissemination of *K. pneumoniae* from the gut to other tissues, including the liver and spleen, with both capsular polysaccharides and the T6SS playing an important role in successful colonization (Calderon-Gonzalez et al., 2023).

*K. pneumoniae* demonstrates notable phenotypic plasticity, allowing it to survive in various host environments. For example, Selective pressures, such as immune responses or nutrient limitations during urinary tract infections, can switch hyper-mucoviscous (hvKP) strains to hypo-mucoviscous forms. This will increase the bacterial adhesion to epithelial cells and support biofilm formation, facilitating chronic colonization while decreasing systemic virulence (Song et al., 2024). OMVs constitute another adaptive strategy utilized by *K. pneumoniae* (Liang et al., 2022). OMVs transport bioactive molecules, like toxins, LPS, and nucleic acids, that modulate host immune responses, promote antibiotic resistance, and facilitate immune evasion. Additionally, OMVs are involved in promoting the dissemination of resistance and virulence factors, as well as horizontal gene transfer within bacterial populations (Lu et al., 2025). *K. pneumoniae*’s genomic plasticity and its accessory genome help in its adaptability and rapid acquisition of mobile genetic elements encoding resistance mechanisms and virulence factors. This genetic variability enhances the pathogens’ ability to evade immune defense and adapt to environmental conditions (Faruk et al., 2024).

Phagocytosis is an important determinant of macrophage-mediated clearance of *K. pneumoniae*. Clinical isolates show differences in susceptibility to macrophage phagocytosis, with hypermucoviscous strains being particularly resistant (van der Geest et al., 2023). Capsule confers resistance to the bacterium by inhibiting recognition by macrophage receptors and preventing opsonization. But the capsule-mutants are readily phagocytosed and are less virulent, highlighting the capsule’s role in immune evasion (Wei et al., 2023). Once internalized, *K. pneumoniae* manipulates phagosome maturation to promote its survival. It inhibits phagosome-lysosome fusion, promoting their replication in vacuolar compartments known as the *Klebsiella*-containing vacuole. This adaptation enables intracellular persistence and protects against lysosomal degradation (Cano et al., 2015).

**Figure 8:**
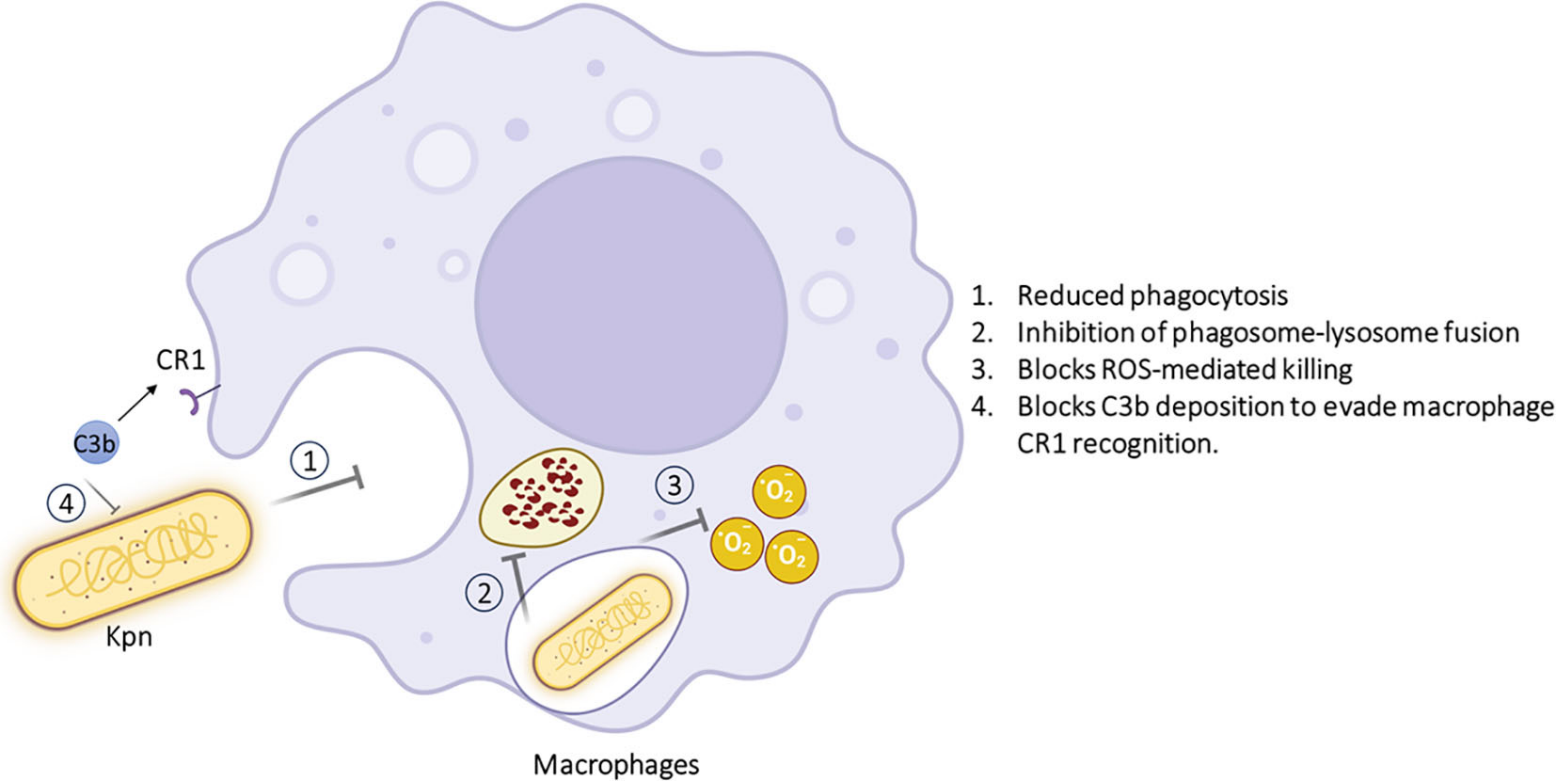
*K. pneumoniae* subverting host innate immune defense. *K. pneumoniae* evades host defenses through multiple strategies, including inhibition of phagosome lysosome fusion, inhibition of phagocytosis, blocks C3b deposition to evade CR1-mediated macrophage recognition and blocking ROS-mediated killing.

Macrophages produce ROS as antimicrobial agents (Tran and Mills, 2024). *K. pneumoniae* resists ROS-mediated killing via antioxidant mechanisms, like its polysaccharide capsule and LPS (Bengoechea and Sa Pessoa, 2019). For example, infections caused by carbapenamase producing *K. pneumoniae* (KPC)-ST258, which are a major cause of healthcare-associated infections worldwide and the polysaccharide part of LPS is responsible for the inhibition of fMLP-induced ROS. *K. pneumoniae* KPC ST258 is a poor inducer of the main bactericidal responses of PMN, ROS generation, and NETs formation compared to another opportunistic Gram-negative bacillus, like *E. coli* (Castillo et al., 2019). These structures neutralize oxidative stress and facilitate bacterial survival within the hostile intracellular environment of macrophages. **Figure 8**, depicts the major immune evasion mechanisms by *K. pneumoniae.* The pathogen modifies macrophages’ cytokine production to facilitate its survival. The CP induces macrophages to produce the pro-inflammatory cytokines, TNF-α and IL-6 (Yang et al., 2011).

The OmpA protein family, a surface-exposed porin, represents a crucial outer membrane protein (OMP) in *K. pneumoniae*. Experimental evidence indicates that deletion of OmpA in *K. pneumoniae* results in increased IL-8 production in bronchial epithelial cells, along with elevated TNF-α and IL-6 levels in mouse lungs. These findings suggest that OmpA may play a significant role in suppressing cytokine secretion by macrophages (March et al., 2011). Additionally, macrophages that are deficient in Regnase-1, a protein that regulates IL-17 and type I interferon responses, exhibit increased resistance to *K. pneumoniae* infection. These macrophages exhibited enhanced type I interferon signaling that promoted better bacterial clearance (Trevejo-Nuñez et al., 2021). Collectively, these demonstrate the potential to modulate cytokine pathways to improve infection outcomes. *K. pneumoniae* alters macrophage cell death pathways, like apoptosis, pyroptosis, to avoid detection by the immune system. By promoting anti-apoptotic signaling via extracellular signal-regulated kinase (ERK) 1/2, K. pneumoniae inhibits macrophage apoptosis, allowing the pathogen to use these cells as a site for replication and dissemination. On the other hand, hypervirulent strains can trigger excessive inflammation that leads to necroptosis, which plays a role in causing tissue damage and disrupting immune function (Wei et al., 2023; Huang et al., 2013).

Classically, microbial pathogens are phagocytosed by macrophages, followed by phagosome–lysosome fusion, bacterial killing, and resolution of infection. This intracellular processing is often accompanied by ROS generation, host cell death, ultimately limiting bacterial survival. However, accumulating evidence indicates that hypervirulent strains of *K. pneumoniae* have been reported to subvert host innate defenses, such as evading phagolysosome maturation and promoting intracellular persistence. Therefore, the discussed evasion strategies enable bacterial survival within host cells and contribute to enhanced pathogenicity and disease severity (Li et al., 2025). Serotype K1/K2 of *K. pneumoniae* strains have been previously reported to be considerably more resistant to phagocytosis than non-K1/K2 strains. Studies have shown that engulfment of the hvKp by macrophages has been significantly lower than the classical *K. pneumoniae* (cKp) (Wang X. et al., 2023). Enhanced autophagy and intact phagosome–lysosome fusion in alveolar macrophages promote intracellular killing; whereas blocking autophagy reduces clearance of *K. pneumoniae* from alveolar macrophages, indicating its survival in the host. Several studies now show that *K. pneumoniae* can turn macrophages into intracellular niches that support replication and dissemination. Most importantly, hypervirulent strains can survive and even replicate within macrophage vacuoles by inhibiting phagosome maturation and preventing fusion with lysosomes, as demonstrated in both *in vitro* macrophage models and *in vivo* tissue macrophages. *K. pneumoniae* interferes with macrophage death pathways, inhibiting apoptosis and modulating autophagy so that infected macrophages remain viable long enough to serve as reservoirs. Capsule polysaccharide and other virulence factors drive anti-inflammatory signaling, generating macrophages that survive, produce regulatory mediators, and thereby create a permissive niche for chronic persistence and abscess formation rather than rapid clearance (Li et al., 2025).

*K. pneumoniae*, mainly hvKp strains, exhibit a remarkable capacity to persist and replicate within macrophages, a process that underpins their ability to form hepatic abscesses. Wanford and colleagues in their study demonstrated that intracellular replication within macrophages is a key mechanism facilitating abscess formation upon hvKp infection (Wanford et al., 2021). Using complementary mouse and pig infection models, along with *in vitro* macrophage and neutrophil assays, they revealed how hvKp subverts innate immune defenses in the liver and resists Kupffer cell-mediated clearance, explaining the strong hepatic tropism of hypervirulent K1 and K2 strains. The authors provided evidence that these hvKp strains could survive and multiply within hepatic as well as in splenic macrophages, forming large intracellular clusters visualized by microscopy. Gentamicin protection assays confirmed their viable intracellular state, and intracellular CFU counts increased over time for hvKp but not for non-hypervirulent isolates, highlighting a specific capacity for resistance to tissue macrophage clearance. However, the molecular mechanisms by which *K. pneumoniae* overcomes macrophage defenses are not fully elucidated. Building on this gap, another group uncovered the mechanisms through which *K. pneumoniae* hijacks host immune signaling. It has been shown that the pathogen exploits type I interferon (IFN) and IL-10 pathways in a TLR4-dependent manner. This promotes STAT6 phosphorylation and leads to the emergence of a distinct macrophage polarization state termed M(Kp). Interestingly, the STAT6-dependent macrophage reprogramming by the pathogen promotes bacterial persistence within macrophages across species, as confirmed in murine, human, and porcine cells. Mechanistic analyses further established that STAT6 activation is essential for *K. pneumoniae* intracellular survival, whereas STAT6 loss *in vivo* facilitates bacterial clearance. Thus, *K. pneumoniae* has evolved to manipulate an evolutionarily conserved innate immune axis, TLR4–type I IFN–IL10–STAT6, to reprogram macrophages in its favor (Dumigan et al., 2022). This anti-immune strategy, driven by the bacterial capsule, differs fundamentally from those used by pathogens such as *Listeria*, *Salmonella*, *Shigella*, or *Mycobacterium*, which rely on direct effector protein delivery to modulate host functions. Moreover, IL-10 production, a hallmark of M(Kp) polarization, has been reported to be governed by the TLR4–MyD88–CREB signaling pathway and is indispensable for intracellular bacterial survival (Li et al., 2025). Together, these studies reveal that *K. pneumoniae* exploits conserved immune signaling pathways to rewire macrophage function, providing a mechanistic basis for its ability to resist clearance and drive abscess formation.

## 9 Conclusion

The Gram-negative opportunistic pathogen *K. pneumoniae* has attracted increasing attention due to the emergence of hypervirulent and antibiotic-resistant strains over the recent decades. The rising prevalence of these isolates has severely restricted, and in some cases eliminated, therapeutic options available for *Klebsiella* infections. This pathogen highlights the disparity between unmet clinical needs and the present research efforts. Indeed, the innate immune cells that defend the host against invading bacteria through myriad defense mechanisms; regrettably, the majority of them are modulated by the pathogens to create a safe niche for their intracellular survival. Recent studies indicate that *Klebsiella* has evolved mechanisms to actively suppress innate immune responses, underscoring the diversity. *K. pneumoniae*’s capacity for both extracellular and intracellular existence, forming the M(Kp), an anti-inflammatory phenotype of macrophages, which exemplifies its sophisticated immune suppression strategies. The intracellular persistence is also promoted by the accumulation of anti-inflammatory metabolites, such as itaconate and IL-10, which inhibit effective bacterial clearance. Investigations into calcium regulation upon *K. pneumoniae* infection might open new insights into host-pathogen interaction and host innate immune suppression mechanisms exploited by the pathogen for intracellular survival. Especially, calcium signaling and homeostasis upon *K. pneumoniae* infection of host cells remain untouched when compared to other human bacterial pathogens.

Moreover, *K. pneumoniae* survives within macrophages by deviating from the canonical endocytic pathway and residing in a distinct intracellular compartment that does not fuse with lysosomes. *K. pneumoniae* manipulates the PI3K–AKT–Rab14 axis to regulate phagosome maturation, indicating its ability to evade and escape from phagocytes. Understanding these mechanisms by which *K. pneumoniae* interacts with immune defenses and modulates metabolic pathways has importance in developing effective treatments. Autophagy and apoptosis generally facilitate bacterial clearance. Surprisingly, *K. pneumoniae* infection induces autophagy and is dependent on the PI3K-AKT-mTOR pathway, and inhibition of this pathway increases the susceptibility of the host to *K. pneumoniae* infection. Autophagy promotion by *K. pneumoniae* can occur through surface recognition or cytoplasmic sensing, although the specific signaling molecules and receptors involved are not fully understood. However, the underlying mechanisms have not been systematically investigated, and numerous knowledge gaps need to be filled in. Identifying the virulence factors involved in host–pathogen interactions, particularly those required for *K. pneumoniae*-induced autophagy, cell death, and cytokine production, is essential. Further studies focusing on the infection biology of *Klebsiella* will enable more precise identification of vulnerable aspects of the immune system and reveal new strategies employed by this pathogen to counteract immune responses.
